# Discovery of suppressors of CRMP2 phosphorylation reveals compounds that mimic the behavioral effects of lithium on amphetamine-induced hyperlocomotion

**DOI:** 10.1038/s41398-020-0753-6

**Published:** 2020-02-24

**Authors:** Wen-Ning Zhao, Brian T. D. Tobe, Namrata D. Udeshi, Lucius L. Xuan, Cameron D. Pernia, Daniel P. Zolg, Amanda J. Roberts, Deepak Mani, Sarah R. Blumenthal, Iren Kurtser, Debasis Patnaik, Irina Gaisina, Joshua Bishop, Steven D. Sheridan, Jasmin Lalonde, Steven A. Carr, Evan Y. Snyder, Stephen J. Haggarty

**Affiliations:** 1 Chemical Neurobiology Laboratory, Center for Genomic Medicine, Massachusetts General Hospital, 185 Cambridge Street, Boston, MA 02114 USA; 2 Departments of Psychiatry & Neurology, Massachusetts General Hospital & Harvard Medical School, Boston, MA 02114 USA; 3 Center for Stem Cells & Regenerative Medicine, Sanford Burnham Prebys Medical Discovery Institute, La Jolla, CA 92037 USA; 4 Department of Psychiatry, University of California San Diego, La Jolla, CA 92037 USA; 5 Sanford Consortium for Regenerative Medicine, La Jolla, CA 92037 USA; 6 Proteomics Platform, Broad Institute of MIT and Harvard University, Cambridge, MA 02142 USA; 7 Department of Medicinal Chemistry & Pharmacognosy, College of Pharmacy, University of Illinois at Chicago, Chicago, IL 60612 USA; 8 Department of Molecular and Cellular Biology, University of Guelph, 50 Stone Road, East, Guelph, ON Canada N1G 2W1; 9 Department of Pediatrics, University of California San Diego, La Jolla, CA 92037 USA; 10 Present Address: Kaiser Health, San Diego, CA USA; 11 Present Address: TUM School of Life Sciences, Technical University of Munich, Munich, Germany; 12 Present Address: Merck, Boston, MA USA

**Keywords:** Predictive markers, Molecular neuroscience

## Abstract

The effective treatment of bipolar disorder (BD) represents a significant unmet medical need. Although lithium remains a mainstay of treatment for BD, limited knowledge regarding how it modulates affective behavior has proven an obstacle to discovering more effective mood stabilizers with fewer adverse side effects. One potential mechanism of action of lithium is through inhibition of the serine/threonine protein kinase GSK3β, however, relevant substrates whose change in phosphorylation may mediate downstream changes in neuroplasticity remain poorly understood. Here, we used human induced pluripotent stem cell (hiPSC)-derived neuronal cells and stable isotope labeling by amino acids in cell culture (SILAC) along with quantitative mass spectrometry to identify global changes in the phosphoproteome upon inhibition of GSK3α/β with the highly selective, ATP-competitive inhibitor CHIR-99021. Comparison of phosphorylation changes to those induced by therapeutically relevant doses of lithium treatment led to the identification of collapsin response mediator protein 2 (CRMP2) as being highly sensitive to both treatments as well as an extended panel of structurally distinct GSK3α/β inhibitors. On this basis, a high-content image-based assay in hiPSC-derived neurons was developed to screen diverse compounds, including FDA-approved drugs, for their ability to mimic lithium’s suppression of CRMP2 phosphorylation without directly inhibiting GSK3β kinase activity. Systemic administration of a subset of these CRMP2-phosphorylation suppressors were found to mimic lithium’s attenuation of amphetamine-induced hyperlocomotion in mice. Taken together, these studies not only provide insights into the neural substrates regulated by lithium, but also provide novel human neuronal assays for supporting the development of mechanism-based therapeutics for BD and related neuropsychiatric disorders.

## Introduction

Treatment of mental illness represents one of the greatest unmet medical needs of our time^1,2^. Despite serious, dose-limiting, adverse effects and lack of efficacy in roughly 60% of patients, the mood stabilizer lithium remains at the forefront of bipolar disorder (BD) treatment due to the absence of better alternatives^3^. Multiple targets of lithium have been discovered, but the capacity of this agent to limit the activity of glycogen synthase kinase-3ß (GSK3β) at therapeutically relevant doses has attracted significant attention^4–8^. This interest is supported by the multiple connections that have been established between dysfunction of GSK3 and the pathophysiology of different neuropsychiatric disorders. Recent human genetic studies, in combination with accumulating evidence from molecular, cellular, pharmacological, and behavioral studies, have consistently recognized the importance of a balance in the activity of GSK3 for mental health^9,10^. In light of these observations, pharmacological control of GSK3 signaling networks is being actively pursued as a potential therapeutic target to treat these severe illnesses.

GSK3 contributes to the regulation of many proteins implicated in a wide range of cellular functions^11–14^. Which GSK3 substrate(s) may be most relevant to the mood and cognitive enhancing properties of GSK3 inhibition by lithium remains unclear and none have been unambiguously associated with the mood and cognitive enhancing properties of GSK3β inhibition. In a recent study, our group revealed an unsuspected link between lithium-dependent GSK3β inhibition and the critical cytoskeleton regulator collapsin response mediator protein 2 (CRMP2, also known as DPYSL2) using patient-derived cellular models of BD^15^. The activity of CRMP2, which is predominantly expressed in the nervous system and exists as either homo- or heterotetramers, can be greatly influenced by multiple post-translational modifications, including phosphorylation, glycosylation, oxidation, and proteolysis^16–18^. Phosphorylation of CRMP2 inactivates it and prompts it to become unbound from the cytoskeletal elements it regulates. As a phosphorylation target of GSK3β, CRMP2 is potentially relevant given its roles in neural development, neuritogenesis, and synaptic function^19–26^. Furthermore, defects in CRMP2 expression and phosphorylation have been reported in relation with many brain disorders, including Alzheimer’s disease^27^, multiple sclerosis^28,29^, Rett syndrome^30,31^, in addition to BD and schizophrenia^32,33^. Hence, it is possible that the regulation of CRMP2 activity by lithium represents a key component of this drug’s efficacy in BD treatment.

Human induced pluripotent stem cell (hiPSC) technology is being used increasingly to create genetically accurate cellular models of diseases, including neuropsychiatric disorders, whether monogenic (e.g., Fragile X syndrome and Rett syndrome^34–36^) or genetically more complex (e.g., schizophrenia^37,38^ and BD^39–41^). In addition to human disease modeling, an exciting opportunity presented by hiPSC technology is the capacity to probe the cellular mechanism of action of drugs used to treat neuropsychiatric disorders and to develop high-throughput phenotypic assays for novel therapeutic discovery^42,43^. For these reasons, we and others have developed neural models through the derivation of homogenous, self-renewing, cultures of neural progenitor cells (NPCs) from hiPSCs^34,44–46^. These hiPSC-derived NPCs can be expanded to provide the sufficient number of cells needed for large-scale high-throughput screening (HTS), an approach successfully implemented for the discovery of WNT signaling pathway modulators^47^. Here, we report the successful use of hiPSC-derived neural cells not only to dissect the effects of lithium on neuronal targets, but also to use that knowledge to develop high-throughput cell-based assays that could, in an unbiased fashion, identify compounds from a library (including FDA-approved drugs) that mimic lithium’s behavioral effects in the amphetamine-induced hyperlocomotion (AIH) test.

## Materials and methods

### Culture and differentiation of hiPSC-derived NPCs into neurons

hiPSC-NPCs derived from a healthy control subject was generated as reported in ref. ^34^ and cultured with expansion media (70% Dulbecco’s modified Eagle medium (DMEM, Invitrogen, Carlsbad, CA, USA), 30% Ham’s F-12 (Mediatech, Manassas, VA, USA), supplemented with B-27 (Invitrogen), 20 ng ml^−1^ each epidermal growth factor (EGF, Sigma-Aldrich, St Louis, MO, USA), and basic fibroblast growth factor (bFGF, R&D Systems, Minneapolis, MN, USA) as previously described in ref. ^47^. Unless mentioned otherwise, the NPC line 8330-8^34^ was used throughout this study. To obtain postmitotic neurons, NPC differentiation was initiated by removal of growth mitogens (EGF, bFGF) from the expansion media and cultures were maintained with medium replacement twice per week. To dissociate and re-plate neuronal cultures differentiated *en masse*, cultures in T75 flasks were washed once with phosphate-buffered saline (PBS), treated with 3 ml of Accutase (Sigma) for 10 min at 37 °C, and then gently triturated several times. Finally, after dilution with fresh media, cells in suspension were passed through a 40 μm cell strainer to remove clumps, counted and spun down. To prepare lysates for western blotting, 1 × 10^6^ cells were seeded per well in 6-well plates. For high-content p-CRMP2 imaging assays, 40,000 cells were seeded per well in 96-well plates (Corning 3904). Re-plated differentiated neuronal cultures were allowed to recover for 1 week before treatment with compounds.

### SILAC (stable isotope labeling by amino acids in cell culture) labeling of NPCs and quantitative mass spectrometry (MS)

NPCs treated with 0, 1, or 10 μM CHIR-99021 were differentially labeled with ‘light’, ‘medium’, or ‘heavy’ SILAC media. In total, two replicates were performed. For the first replicate the 0 μm CHIR-99021 treatment was labeled with heavy media, the 1 μM treatment with light media, and the 10 μM treatment with medium media, while in the second replicate the 0 μM treatment was labeled with light media, the 1 μM treatment with medium media, and the 10 μM treatment with heavy media. Complete description of SILAC labeling and subsequent quantitative analysis of protein phosphorylation is available in the supplemental information of our previous publication^15^. For the purpose of this manuscript, mass spectrometry data from this previous publication was reprocessed using an updated human reference proteome UniProt database (release 2014_10) subtracting a set of 150 common laboratory contaminant proteins with Spectrum Mill software (Agilent Technologies). Similar MS/MS spectra acquired on the same precursor *m*/*z* within ±60 s were merged. MS/MS spectra were excluded from the analysis if they were not within the precursor MH+ range of 600–4000 Da, or if they failed the quality filter by not having a sequence tag length >0. Spectra were allowed ±20 p.p.m. mass tolerance, 30% minimum matched peak intensity, and ‘trypsin allow P’ enzyme specificity with up to four missed cleavages. The fixed modifications were carbamidomethylation at cysteine and SILAC: Arg (6–10 Da), Lys (4–8 Da) mix. Allowed variable modifications were phosphorylation of serine, threonine, and tyrosine residues, oxidized methionine, pyroglutamic acid on N-termini glutamines, and acetylation of protein N-termini with a precursor MH+ shift range of −18 to 258 Da. Individual spectra were automatically assigned a confidence score using Spectrum Mill autovalidation module. A target–decoy FDR of 1.2% was used. A Spectrum Mill Protein-Var Mod Site Comparison was generated to identify only phosphopeptides. For data analysis, phosphopeptides not observed in both SILAC experiments and decoy peptides were removed. In addition, any phosphopeptides that did not have at least 1 fully localized phosphorylation site were removed, and any phosphopeptides not quantified in both replicates for at least 1 condition of CHIR-99021 treatment were excluded. Finally, ratios were median normalized and transformed into log2 space. Protein–protein interaction network modeling of differentially regulated phosphoproteins was performed using VisANT 5.0, a freely available (http://visant.bu.edu) network modeling software program^48^.

### Western blotting

Protein lysates for western blot analyses were prepared from NPCs or 4-week differentiated human neuronal cultures in 6-well plates treated with GSK3 inhibitors at the indicated concentrations for 6 or 24 h. Western blotting was performed using 4–12% Bis-Tris pre-cast mini gels (Invitrogen) for electrophoresis, PVDF membrane for protein transfer, and overnight incubation at 4 °C for primary antibodies. The antibodies recognizing p-CRMP2^T514^ (#9397, 1:1000), and total CRMP2 (#9393, 1:1000) were purchased from Cell Signaling Technologies (Beverly, MA, USA).

### Immunocytochemistry (ICC)

Fluorescence ICC was performed according to procedures previously described^47^. The following primary antibodies were used to assess neuronal differentiation: TuJ1 (T8660, Sigma), MAP2 (CPCA-MAP2, EnCor Biotechnology, Gainesville, FL, USA), SMI312 (SMI-312R, Covance, Princeton, NJ, USA), and GFAP (MCA-5C10; EnCor Biotechnology). To evaluate CRMP2 expression and phosphorylation levels with ICC, the same p-CRMP2^T514^ and CRMP2 antibodies from Cell Signaling Technologies as for western blot analysis were used. For the high-throughput high-content screening assay, neuronal cultures were co-immunostained for p-CRMP2^T514^, the dendritic marker MAP2, and the nuclear protein HDAC2 (05-814, EMD Millipore, Burlington, MA, USA).

### High-throughput neuronal assay for p-CRMP2^T514^

With the exception of lithium, which was added manually, compounds were applied to 4-week differentiated human neuronal cultures prepared in 96-well plates using a CyBio Well vario automated liquid handling system equipped with a 96-pin head (Analytik Jena AG, Jena, Germany). More precisely, 100 nl of each compound solubilized in DMSO and arrayed in compound plates was transferred to 96-well assay plates with 100 μl culture media per well. After a period of 24 h, unless specified otherwise, cells were fixed for 30 min at room temperature with 4% paraformaldehyde (PFA) in PBS, washed with PBS to remove PFA, and then immunostained for p-CRMP2^T514^, MAP2 (neuronal marker), and HDAC2 (nuclear marker). Immunostained neurons were either scanned on a laser-scanning cytometer (Acumen eX3, TTP Labtech, Cambridge, MA, USA), with a 488 nm laser setting of 100% power and 550-volt detection setting, or imaged with an IN Cell Analyzer 6000 automated confocal microscope (GE Healthcare Life Sciences, Marlborough, MA, USA) for high-content analysis. Data are expressed as mean ± SEM of at least three replicates. Unpaired *t*-tests were used to determine treatment significance for all compounds tested. Stars of significance indicate a significant effect for a treatment dose compared with control (*0.01 ≤ *p* < 0.05, **0.001 ≤ *p* < 0.01, ***0.0001 ≤ *p* < 0.001, ****p < 0.0001).

### High-content image acquisition and analysis

High-content images were acquired using an IN Cell Analyzer 6000 automated confocal microscope (GE Healthcare Life Sciences, Marlborough, MA, USA) with a ×20 objective. A total of 4–6 fields of images from each well of 96-well plates were collected. Image analysis was performed with the IN Cell Investigator Software v1.6.2. Precisely, after identification of all nuclear objects, a collar ring was drawn around each nucleus and filters were then applied to identify MAP2-positive neurons in the mixture of cells according to the following analysis criteria: (1) size of nuclear objects: too small (<50 μm)—cell debris or too large (>160 μm)—cell clumps, (2) intensity of nuclear staining: too dim (<2197 pixels)—falsely identified objects, (3) intensity of MAP2 staining in the collar ring: low MAP2—not neurons, and (4) coefficient variation (CV) of MAP2 staining in the collar ring: CV > 1—neurons. Neurites were traced out from all nuclei on the basis of the MAP2 signal, and both p-CRMP2^T514^ intensity and MAP2 intensity measured from identified neurites. From population of MAP2-positive neurons, measured p-CRMP2^T514^ and MAP2 intensities, reported as averages for each well, were used to calculate percentage of p-CRMP2^T514^ to MAP2. Finally, the number of MAP2-positive neurons was used to evaluate toxicity of compounds.

### Amphetamine-induced hyperlocomotion test of lithium-responsive behavior in mice

AIH assays were performed as described in Gould et al.^49^. The following compounds were administered to mice either intraperitoneally (IP), subcutaneously (SC), or by gavage (per os, PO) in a volume of 0.01 ml/g body weight as following: methamphetamine 3.5 mg/kg, IP in saline (Sigma-Aldrich); LiCl 85 mg/kg, IP in saline (Sigma-Aldrich); ropinirole 10 mg/kg, IP in dH_2_O (Sigma-Aldrich); oxiracetam 100 mg/kg, IP in dH_2_O (BOC Sciences, Shirley, NY, USA); Huperzine A 1 mg/kg, PO in dH_2_O with 40% volume 0.1 M HCl (Sigma-Aldrich); sulpiride 10 mg/kg, IP in saline with three drops of Tween-80 (Tocris Bioscience, Bristol, UK); ganaxolone 10 mg/kg, IP in 20% 2-hydroxypropyl-beta-cyclodextrin (Tocris Bioscience); forskolin 1 mg/kg, SC suspended in 10% cremaphor (Tocris Bioscience); NNC-711 5 mg/kg, IP in saline and sonicated (Tocris Bioscience). A cohort of 32 C57BL/6J mice and another one of 32 DBA/2J mice were used to test huperzine A, oxiracetam, and ropinirole. A third cohort of 32 C57BL/6J mice was used to test forskolin, ganaloxone, NNC-711, and sulpiride. Each cohort was tested once per week. For statistical analyses, the effect of compounds was initially analyzed by two-way ANOVA’s between-subjects factors lithium (vehicle or lithium) and compound (vehicle or compound). In addition, between-group analysis of separate cohorts of six mice per group was used in each separate experiment to test the effect of sulpiride pretreatment on AIH also in comparison with vehicle, lithium, and lithium plus sulpiride. For group allocation, there was no blinding and randomization.

## Results

### Profiling of GSK3β neural substrates in hiPSC-derived NPCs with SILAC

To address which phosphorylation sites in the human neural proteome are under the control of GSK3, we first set up to identify candidate targets that are directly changed by pharmacological inhibition of GSK3 in hiPSC-derived NPCs using quantitative SILAC-based mass spectrometry (MS). To inhibit GSK3 with high selectivity and efficacy for this MS-based quantitative proteomics screen, we chose to use the cell-permeable, ATP-competitive, aminopyrimidine derivative CHIR-99021, which is recognized as one of the most selective and potent inhibitors of GSK3 identified to date^8^. Importantly, although comparative analysis of the two key regulatory GSK3 phosphorylation sites (using GSK3β numbering), tyrosine 216 (Tyr216) and serine 9 (Ser9)^50–52^ (Supplemental Fig. 1a), in hiPSC-derived NPCs and neurons suggested different response patterns to CHIR-99021 and lithium treatments (Supplemental Fig. 1), we found that both agents, although with different potencies, had a very similar impact on downstream GSK3-dependent WNT signaling gene expression (Supplemental Fig. 2). The difference between CHIR-99021 and lithium in terms of resulting GSK3 phosphorylation can be explained by the fact that Tyr216 is regulated by autophosphorylation^53^ and is critically required for activity of GSK3, while, on the other hand, increasing levels of Ser9 phosphorylation results in inhibition of the kinase activity (termed inhibitory phosphorylation) that is dominant to effects of Tyr216 phosphorylation^52,54^. Therefore, in addition to lithium directly inhibiting GSK3 kinase activity through competitive inhibition of Mg^2+^ at the ATP-binding site, but not to ATP itself or substrates^55^, its targeting of molecular events contribute to an indirect inhibition of kinase activity through augmentation of GSK3 Ser9 phosphorylation in an AKT-dependent manner that leaves Tyr216 largely unaffected^6,8,56^. In contrast, CHIR-99021 limits phosphorylation of residue Tyr216 via directly altering the intrinsic kinase activity of GSK3 by blocking the kinase’s ATP-binding site, although an effect of increasing dephosphorylation through an indirect phosphatase-mediated mechanism that lithium does not invoke cannot be ruled out. In either case, the result is a comparable diminution in the levels of GSK3 substrate phosphorylation.

**Fig. 1:**
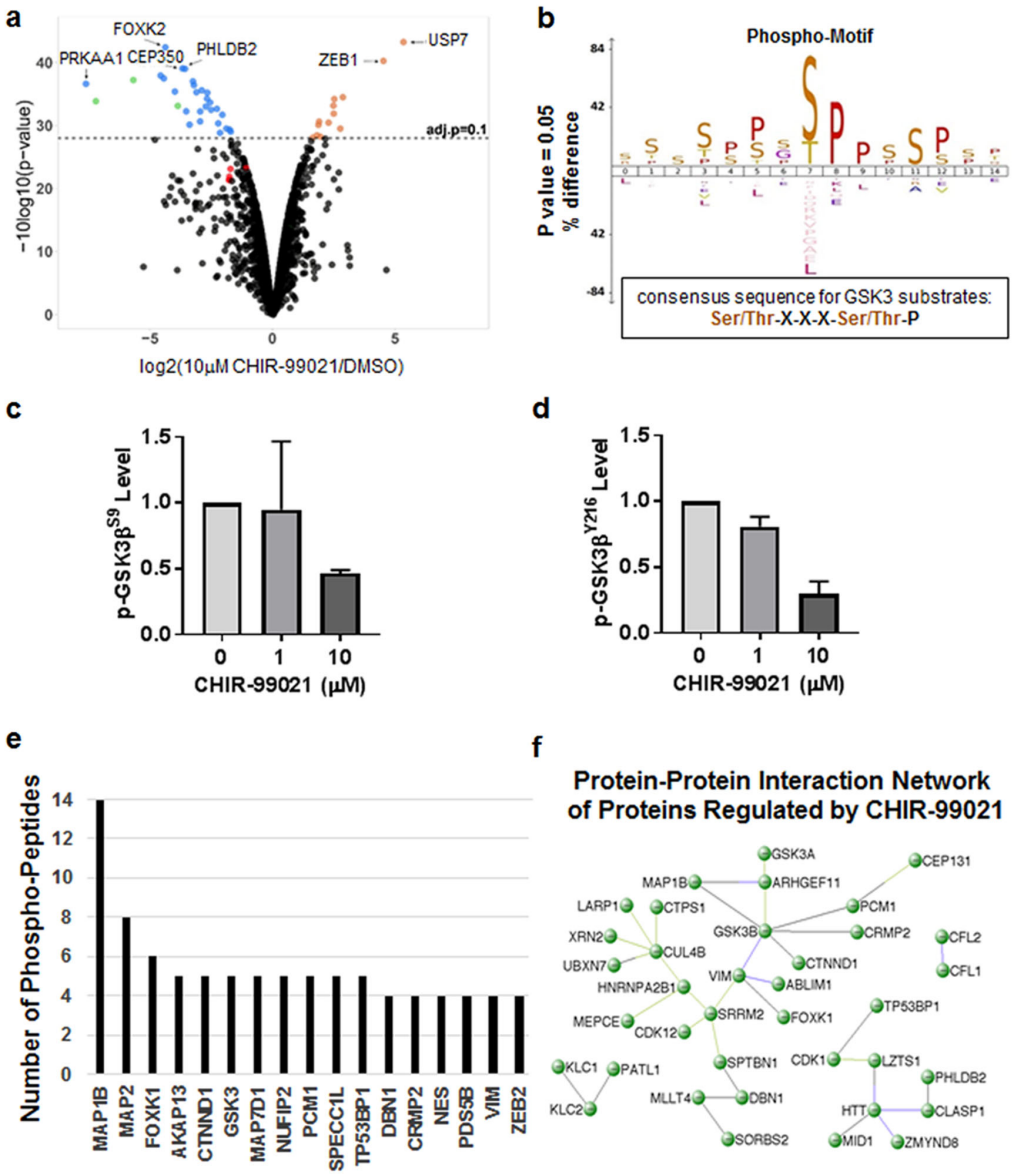
Discovery of candidate GSK3 neural substrates with SILAC phosphoproteomics. **a** Volcano plot of phosphopeptides identified in the 10 μm CHIR-99021 treatment. Fold changes are plotted versus the −log_10_ of their *P*_nom_ values. Out of 2829 phosphopeptides, 43 were significantly regulated by 10 μM CHIR-99021 (dots above dashed line—blue and brown dots). Green dots represent phosphopeptides mapped to CRMP2 protein, while red dots to GSK3α/β. **b** Phospho-motif enriched in phosphopeptides downregulated by 10 μm CHIR-99021 (weblog.berkeley.edu). The phospho-motif matches the consensus sequence of GSK3 substrates, which is Ser/Thr-X-X-X-Ser/Thr-P where the first Ser or Thr is the target residue, X is any amino acid (but often Pro), and the last Ser-P/Thr-P is the site of priming phosphorylation. **c**, **d** Graphs show the ratios of p-GSK3β^S9^ (**c**) and p-GSK3β^Y216^ (**d**) in CHIR-99021-treated over control. Note that the phosphopeptides for GSK3β are shared with GSK3α and therefore have been labeled as such in Supplemental Table 1. **e** Top 17 proteins most significantly altered by CHIR-99021, which are represented by at least 3 or more regulated phosphopeptides. **f** Protein–protein interaction network of proteins regulated by CHIR-99021, visualized using VisANT (5.0). Thirty-seven proteins that had at least one interaction with another protein formed three submodules centered around GSK3β, HTT, and KLC2.

Building on our previous SILAC-MS dataset^15^ by re-analysis of the peptide level data through an updated computational pipeline and the latest version of the UniProt human proteome database, including removal of a set of 150 common laboratory contaminant proteins, a complete list of all high confidence, phosphopeptides identified by SILAC-MS upon treatment of hiPSC-derived NPCs with two concentrations of CHIR-99021 (1 or 10 μM) or vehicle control (DMSO) was generated (Supplemental Table 1). In total, this new analysis identified 2829 phosphopeptides of which 43 were significantly regulated in the CHIR-99021 (10 μM) treatment after correction for multiple hypothesis testing (Fig. 1a). Phosphopeptides significantly downregulated (*p* < 0.05) upon CHIR-99021 (10 μM) treatment were used to confirm the sequence of the enriched phospho-motif (Fig. 1b). As expected, but demonstrated experimentally for the first time with human neuronal cells treated with a GSK3 inhibitor, the phospho-motif identified matched the canonical consensus sequence of GSK3 substrates, which corresponds to Ser/Thr-X-X-X-Ser/Thr-P where the first Ser or Thr is the target residue, X is any amino acid, and the last Ser-P/Thr-P is the site of priming phosphorylation^57^. In addition, these phosphoproteomic data showed that phosphorylation of GSK3β at both Tyr216 and Ser9 was decreased as a result of CHIR-99021 treatment (Fig. 1c, d), which was consistent with our western blot analyses (Supplemental Fig. 1). As the phosphopeptides for GSK3β were shared with GSK3α they were therefore noted as such in Supplemental Table 1.

Having validated the global change in the phosphoproteome maps to the known GSK3 phospho-motif, we next performed a global, systems-level analysis of the change in the neural phosphoproteome upon CHIR-99021 treatment. The total of 575 peptides were identified as differentially regulated (*p* < 0.05) upon CHIR-99021 (10 μM) treatment mapped to 398 unique proteins with 37 proteins (grouping GSK3α/β into one protein) represented by at least 3 or more regulated phosphopeptides as shown in Fig. 1e. The top regulated protein upon CHIR-99021 treatment was the microtubule-associated protein 1 B (MAP1B) with a total of 14 phosphopeptides followed next by microtubule-associated protein 2 (MAP2) with 8 phosphopeptides, both of which are involved in cytoskeletal dynamics and have been previously shown to be GSK3 substrates in multiple studies. The third most regulated protein was the Forkhead box protein 1 (FOXK1) transcription factor implicated in both metabolic gene regulation downstream of mTOR and WNT signaling through DVL1^58,59^, which is also known to be a GSK3 substrate.

Having identified which individual proteins were most significantly altered by CHIR-99021 treatment, as well as verified that multiple of these were known substrates of GSK3, we next sought to create an integrative, systems-level understanding of the signaling networks affected by this treatment. For this computational analysis we considered a total of 90 unique proteins with at least 2 or more phosphopeptides among the set of 575 differentially regulated phosphopeptides (*p* < 0.05) upon CHIR-99021 (10 μM) treatment and constructed an interaction network to visualize their known protein–protein interactions using VisANT (5.0)^48^. The resulting network graphs shown in Fig. 1f comprised 37 proteins (41%) that had at least one interaction with another protein in the set. In total there were 3 submodules with a main connected component comprising 24 nodes centered around GSK3β with 6 interactions and CUL4B, an E3 ubiquitin ligase component, with 5 interactions. The second largest submodule centered around the Huntingtin (HTT) protein contained 8 proteins, the third submodule centered around KLC2 had 3 proteins, and lastly a 2-protein module with interactions between the two cofilin family members CFL1 and CFL2 involve in actin cytoskeleton regulation. Taken together, these global proteomic data provide evidence that, besides proteins with known protein–protein interactions with GSK3, multiple signaling networks—many dealing with cytoskeleton function—are regulated upon inhibition of GSK3 kinase activity.

Finally, we performed multiplexed gene expression profiling using the L1000 assay and demonstrated regulation of multiple components of the WNT signaling pathway shared between CHIR-99021, lithium, and Wnt3a treatment in the same dose range and time period of treatment as performed for our phosphoproteomic experiment (Supplemental Fig. 2). These data support the notion that CHIR-99021-sensitive phosphorylation changes can be translated into functional effects on cellular pathways known to be regulated by lithium in hiPSC-derived NPCs.

### Phosphorylation of the cytoskeletal regulator CRMP2 at Thr514 is decreased by CHIR-99021 and lithium

**Fig. 2:**
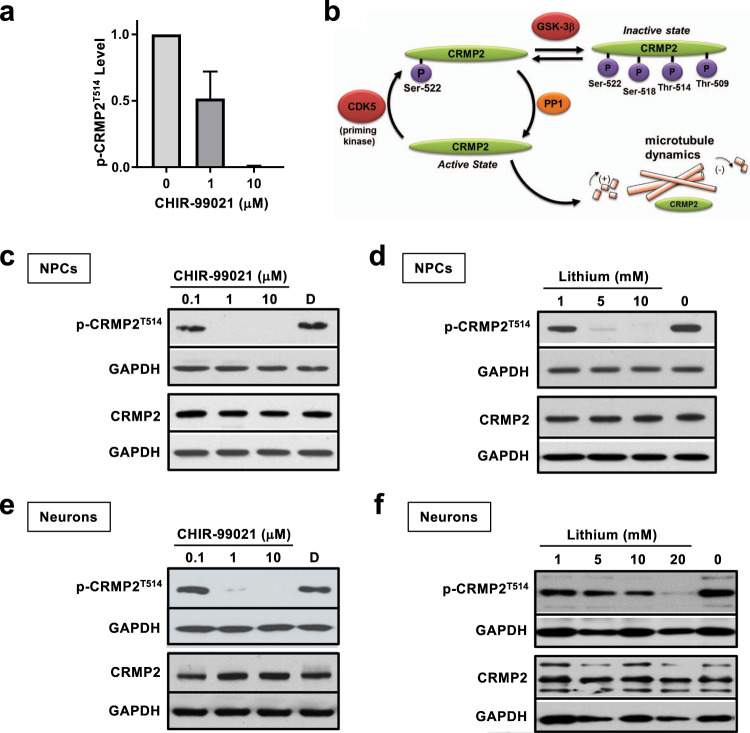
Reduced phosphorylation of CRMP2 at Thr514 upon treatment with CHIR-99021 and lithium. **a** CRMP2 was identified with reduced phosphorylation at Thr514 as previously reported in Tobe et al.^15^. A bar graph of CRMP2^T514^ phosphopeptide ratios in CHIR-99021-treated over control is shown. The UniProt gene symbol for CRMP2 is DPYSL2. **b** Schematic of key phosphorylation events on CRMP2 involving CDK5 and GSK3β. CDK5 phosphorylates CRMP2 at Ser522, providing a primed substrate for subsequent phosphorylation by GSK3 at Ser518, Thr514, and Thr509. Phosphorylation of CRMP2 at Thr514 has been shown to be GSK3β-specific. The phosphorylation events are essential for diverse CRMP2 cellular functions. **c** Western blot analysis validated that p-CRMP2^T514^ levels were decreased after 6-h treatment of NPCs with CHIR-99021 in a dose-dependent manner. Total CRMP2 protein was not affected by the treatments. **d** Lithium treatment in NPCs for 6 h effectively reduced p-CRMP2^T514^ levels in a dose-dependent manner. **e** CHIR-99021 and **f** lithium also exhibited dose-dependent reduction of p-CRMP2^T514^ on the 4-week differentiated neuronal cultures. Total CRMP2 protein was not affected by the treatments.

Our re-analysis of the mass spectra and consideration of the proteins represented by at least 3 or more regulated phosphopeptides upon CHIR-99021 treatment as shown in Fig. 1e confirmed our original observations that 24 h treatment with CHIR-99021 at either 1 or 10 μM robustly reduced phosphorylation of multiple CRMP2 phosphopeptides without changing total CRMP2 protein levels (Fig. 2a)^15^. Many of CRMP2’s diverse cellular functions are extensively regulated by phosphorylation, most notably by the cyclin dependent kinase 5 (CDK5) at serine 522 (Ser522), a necessary priming step, and subsequent phosphorylation by GSK3 at Ser518, Thr514 and Thr509^25,60–62^, resulting in inactivation of CRMP2’s tubulin-binding properties that regulates microtubule assembly (Fig. 2b). Of note, characterization of selective knockout of GSK3 isoforms and biochemical studies with recombinant GSK3α and GSK3β suggest that CRMP2 phosphorylation at Thr514 is specifically regulated by GSK3β and not GSK3α thereby providing a functional marker of GSK3β target engagement^63^.

To confirm and extend these MS-based proteomic findings with CHIR-99021, and to determine if they could be also observed with the much less potent, but clinically relevant mood stabilizer and GSK3 inhibitor lithium, NPCs were treated with different doses of CHIR-99021 or lithium for 6 or 24 h and western blotting performed on total cell lysates with a phosphospecific antibody that recognizes CRMP2 when phosphorylated at Thr514 (p-CRMP2^T514^). Robust reduction was detected from 6-h treatment samples at the lowest dose tested for both CHIR-99021 (0.1 μM) and lithium (1 mM) with a complete reduction at higher concentrations, whereas the total amount of CRMP2 protein did not change (Fig. 2c, d). In other words, the p-CRMP2^T514^:total-CRMP2 ratio was decreased. Similar results were obtained for 24-h treatment (data not shown). Together, these results validated the original MS-based proteomic findings using an orthogonal, antibody-based detection method and indicate that p-CRMP2^T514^ is highly sensitive to GSK3 inhibitor treatment in hiPSC-derived NPCs. Moreover, the observation of the effect of lithium at therapeutically relevant doses suggests CRMP2 phosphorylation may be relevant to the mood stabilizing properties of GSK3 inhibitors as well as lithium.

We further examined the effect of CHIR-99021 and lithium treatments on p-CRMP2^T514^ levels in neurons by western blotting (Fig. 2e, f). Four-week differentiated, 24-h treated hiPSC-derived neurons were probed for p-CRMP2^T514^ or total CRMP2. Dose-dependent effects of reduced p-CRMP2^T514^ were observed for CHIR-99021 and lithium. A trace amount of p-CRMP2^T514^ was detected at 1 μM CHIR-99021 whereas no band detectable at 10 μM. For lithium, p-CRMP2^T514^ steadily decreased upon the increase of concentrations (1, 5, 10, 20 mM) and the band was nearly not visible at 20 mM treatment.

### Phosphorylation of CRMP2 at Thr514 is decreased by GSK3 inhibitors with different chemical structures

To extend the observations with CHIR-99021 and lithium, we next examined several existing GSK3 inhibitors that have distinct chemical structures and cover a range of GSK3 inhibitory activities (Supplemental Fig. 3a). hiPSC-derived NPCs treated with three different concentrations (0.1, 1, 10 μM) of each compound for 24 h were collected and the amount of p-CRMP2^T514^ in each lysate was evaluated by western blotting (Supplemental Fig. 3b). All four GSK3 inhibitors tested decreased p-CRMP2^T514^ levels in a dose-dependent manner and the magnitude of effects matched previously reported GSK3 inhibitory activities, with CHIR-99021 being the most potent with noticeable effect at 0.1 μM, and AR-A014418 only showing decreased p-CRMP2^T514^ levels at a much higher dose (10 μM).

Next, we decided to further examine the effect of various GSK3 inhibitor treatments on p-CRMP2^T514^ but in hiPSC-derived differentiated neurons (Supplemental Fig. 3c). Consistent with our NPC results, the most potent GSK3 inhibitor among the group, CHIR-99021, presented the strongest effect on p-CRMP2^T514^ level, whereas the weakest, AR-A014418, elicited measurable decrease only at the highest concentration tested, which indicates that the potency of these known GSK3 inhibitors is reflected by the level of detectable p-CRMP2^T514^. Together, these data demonstrate that p-CRMP2^T514^ levels provides a robust readout of GSK3 kinase activity in human iPSC-derived NPCs and neurons.

Many but not all GSK3 substrates require priming phosphorylation of three amino acids adjacent to the GSK3’s phosphorylation sites. In the case of CRMP2, CDK5 is known to phosphorylate CRMP2 at Ser522, priming it for subsequent phosphorylation by GSK3β that to preferentially phosphorylates Ser/ThrXXX(pSer/Thr) motifs, where X is any residue^62,64^. In order to test whether CDK5 activity plays a role in priming CRMP2 phosphorylation, NPCs were treated with the CDK5 inhibitor roscovitine using a stock that had been validated for activity using an in vitro CDK5 enzymatic assay (data not shown). Unexpectedly, treatment with roscovitine had no evident effect on p-CRMP2^T514^ or p-CRMP2^S522^ levels (Supplemental Figs. 3b, c and 4). This result suggests that either the phospho-priming of p-CRMP2^S522^ is regulated by kinases other than CDK5 or that the majority of CRMP2 is already phosphorylated by CDK5 in NPCs without sufficient turnover over the duration of the experiment to reveal a detectable effect. However, although we validated the same stock of roscovitine used in these cellular assays for its activity using an in vitro CDK5 enzymatic assay (data not shown), and a similar concentration range (1–10 μM) was demonstrated to reduce p-CRMP2^S522^ levels in rat NPCs^65^, since our experiments lacked a positive control for intracellular CDK5 inhibition other than p-CRMP2^S522^ we cannot rule out insufficient CDK5 target engagement as an additional explanation for the lack of observed effect.

In an effort to expand the panel of GSK3 inhibitors tested for their ability to regulate p-CRMP2^T514^ levels in human neurons, we also used our recently discovered novel series of pyrazolo-tetrahydroquinolinone GSK3 inhibitors (Supplemental Fig. 3d)^66,67^. Previous work has shown that chiral compound JB1121(*ent-2*)/BRD3937 conveys selective and potent inhibition of GSK3^66,67^. Having established cellular assays in hiPSC-derived NPCs that can report on GSK3 inhibition^47^, and given that this compound series had not previously been tested in non-transformed human cell lines, we first tested the activities of the racemic compound JB1121(*rac*) and its two separated enantiomers for their ability to stimulate canonical WNT signaling pathways and promote NPC proliferation. Notably, JB1121(*ent-2*) increased WNT signaling reporter activity at 12.5 and 25 μM (Supplemental Fig. 5a), and produced a dose-dependent increase of NPC proliferation starting at ~0.5 μM (Supplemental Fig. 5b). In contrast, JB1121(*ent-1*) failed to show activity. In both assays, JB1121(*ent-2*) produced higher activity than the racemic mixture at each concentration. As a more target-focused, neural assay we next tested whether these novel GSK3 inhibitors could modulate p-CRMP2^T514^ levels. Three concentrations (0.1, 1, 10 μM) of JB1121(*rac*) and separated enantiomers were used to treat hiPSC-derived NPCs (Supplemental Fig. 3e) or neurons (Supplemental Fig. 3f) for 24 h, after which p-CRMP2^T514^ and CRMP2 expression was examined by western blotting. Here, JB1121(*rac*) and JB1121(*ent-2*) effectively reduced p-CRMP2^T514^ in human NPCs and neurons in a dose-dependent manner whereas JB1121(*ent-1*) was inactive even at the highest concentration (10 μM) tested. In summary, cellular activities obtained in reducing p-CRMP2^T514^ in the hiPSC-derived NPCs or neurons for GSK3 inhibitors across multiple structurally distinct series correlate well with their in vitro GSK3 inhibitory activities.

### High-throughput phenotypic assay for suppressors of p-CRMP2^T514^

Considering the interest of adapting patient-specific, hiPSC-derived human neurons for high-throughput screens (HTS), we sought to develop a human neuron-based functional assay for GSK3β inhibitors through quantification of p-CRMP2^T514^ levels. Based on an extended set of assay development and refinements, we used human neurons differentiated for 4 weeks with growth factor withdrawal that showed a robust degree of morphological and marker-specific differentiation (Supplemental Fig. 6a). Under these conditions, we first examined the expression of p-CRMP2^T514^ by immunocytochemistry (Supplemental Fig. 6b). Immunoreactivity of total CRMP2 and p-CRMP2^T514^ was detected on all processes in addition to the soma, consistent with previous findings that CRMP2 is involved in axonal guidance and dendritic field organization^19,68,69^.

**Fig. 3:**
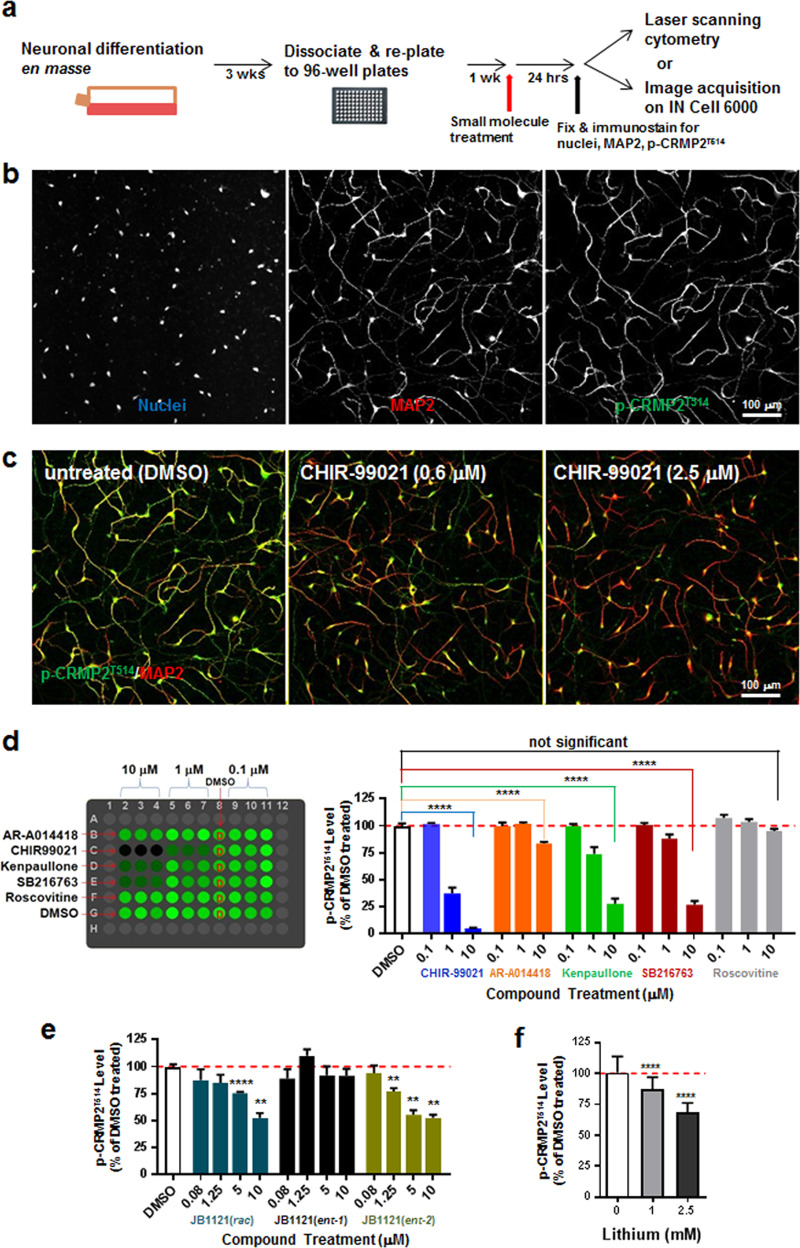
High-throughput phenotypic assay using human iPSC-derived neurons for modulators of CRMP2 phosphorylation. **a** High-throughput assay procedures, in which expanded NPCs were induced into postmitotic differentiation by growth factor withdrawal in flasks, maintained with periodic media change for 3 weeks, at which time the neuronal culture was dissociated, re-plated to 96-well plates, and matured for another week before small molecule compound treatment for 24-h followed by fixation, immunofluorescence staining, and detection of p-CRMP2^T514^ signal intensity using laser-scanning cytometry (Acumen eX3) or automated confocal microscopy for image acquisition followed by subsequent image analysis. **b** Human neuronal cultures immunostained for nuclei, MAP2 and p-CRMP2^T514^. Postmitotic neurons differentiated from hiPSC-derived NPCs for four weeks were seeded in 96-well plates for treatments and immunocytochemistry. **c** Representative images of CHIR-99021 treatment on p-CRMP2^T514^ intensity (green) versus MAP2 intensity (red). Intensity of p-CRMP2^T514^ decreases when CHIR-99021 concentration increases. **d** p-CRMP2^T514^-reducing activities from existing GSK3 inhibitors shown by heatmap and quantification of p-CRMP2^T514^ levels. Dose-dependent activities correspond well to their GSK3 inhibitory activities. **e** Quantification of p-CRMP2^T514^ levels for the GSK3 inhibitor JB1121(*rac*) and its enantiomers. JB1121(*ent-2*) but not JB1121(*ent-1*) showed dose-dependent effects. **f** Dose-dependent effect of lithium reducing p-CRMP2^T514^ detected in the human neuronal assay. Relative p-CRMP2^T514^ intensity level was reported as the percentage of the intensity for DMSO-treated (100%). Unpaired *t*-test: **0.001 ≤ *p* < 0.01, ****p < 0.0001.

Given the strong correlation of p-CRMP2^T514^ level with the potency of selected GSK3 inhibitors and compound concentrations by western blotting (Supplemental Fig. 3), we next confirmed this correlation with immunocytochemistry in order to establish a high-content, image-based screening assay in neurons (Fig. 3a–c). To achieve this, arrayed prototypical GSK3 inhibitors (0.1, 1, and 10 μM) along with DMSO as the vehicle were robotically pin-transferred into 96-well plates with neurons. After a 24-h treatment, cells were fixed and immunostained for nuclei (HDAC2), MAP2 (dendrites) and p-CRMP2^T514^ (Fig. 3b) with a representative image from CHIR-99021-treated samples demonstrating decreasing p-CRMP2^T514^ shown in Fig. 3c. Next, the plates were screened on a laser-scanning cytometer to assess the relative level of p-CRMP2^T514^ signal intensity. As shown in Fig. 3d, the prototypical GSK3 inhibitors exhibited varying degrees of p-CRMP2^T514^ reduction and the CDK inhibitor roscovitine did not show any detectable effect, consistent with previous observation on western blot analysis (Supplemental Fig. 3b, c). To further evaluate the robustness of the assay, we performed a time-course experiment where neurons were treated for 3, 6, or 24-h. Using this approach, we found that even with a 3-h treatment, the activity of prototypical GSK3 inhibitors with moderate potency could be captured by the immunofluorescence detection of p-CRMP2^T514^ (Supplemental Fig. 7). After this validation of the p-CRMP2^T514^ laser-scanning cytometry assay with prototypical GSK3 inhibitors, we finally tested the JB1121 series of highly selective GSK3 inhibitors at several concentrations (0.08, 1.25, 5, and 10 μM) (Fig. 3e)^66,67^. A dose-dependent decrease of p-CRMP2^T514^ levels was detected for JB1121(*R*) as well as racemic JB1121(*S*/*R*) confirming the capability of the assay to report on highly selective GSK3 inhibitors in addition to CHIR-99021.

To further improve on the sensitivity of the laser-scanning cytometry assay, we next adapted the p-CRMP2^T514^ immunofluorescence detection to a high-content, single-cell level assay by collecting high-resolution images on an automated confocal microscope (Fig. 3a). A custom image analysis pipeline was subsequently applied to identify well-defined neurons in the heterogeneous culture, and finally levels of p-CRMP2^T514^ and MAP2 were measured to evaluate the effect of compounds at the level of individual neurons (Supplemental Fig. 8). The sensitivity of the high-content, single-cell assay was validated by treating the neuronal culture with lithium, which caused reduction of p-CRMP2^T514^ levels to 68.4% or 87.2% of control for 2.5 or 1 mM lithium (Fig. 3f), respectively.

### High-content, single-cell-based chemogenomic screen for suppressors of p-CRMP2^T514^

**Fig. 4:**
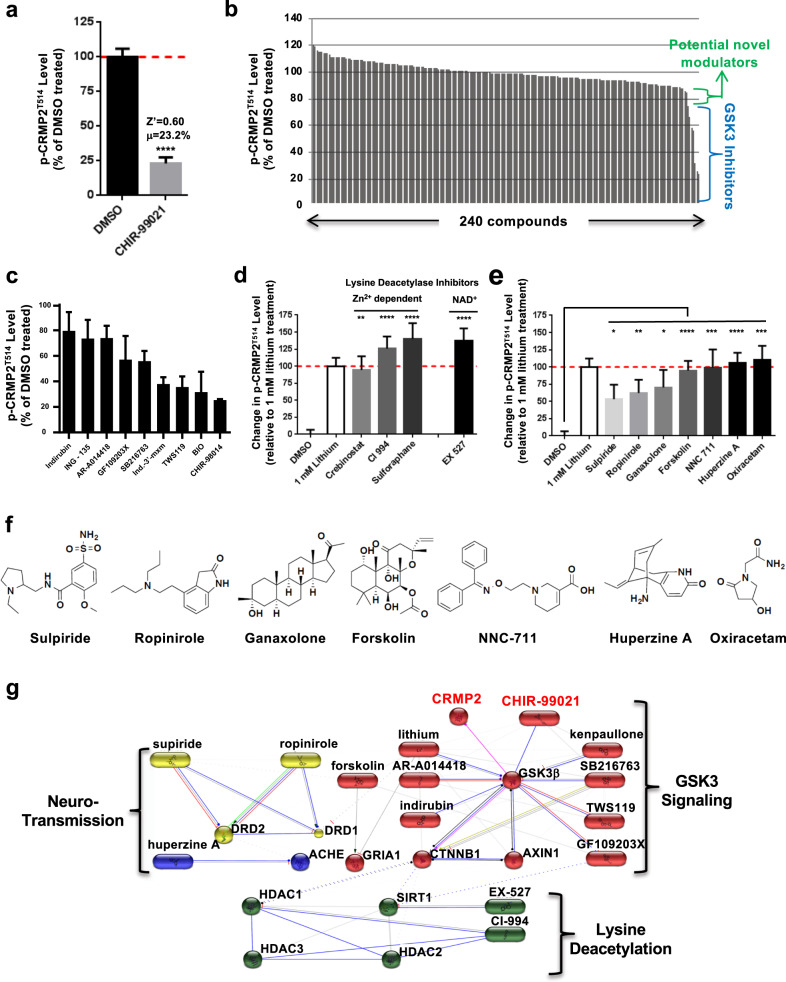
High-content screen for CRMP2-phosphorylation modulators. **a** Z′ factor = 0.6 was calculated with 10 μM CHIR-99021 used as positive control in the high-content screen. **b** High-content screen of 240 compounds for p-CRMP2^T514^ modulators (*n* = 3 replicates). GSK3 inhibitors are most effective modulators down-regulating p-CRMP2^T514^ levels to below 80%, indicated by blue bracket. Smaller effect is detected for a group of potential hits (green bracket). **c** Graph of the data for GSK3 inhibitors from the screen. **d** Re-test of a group of novel modulators that are lysine deacetylase inhibitors. Crebinostat, a novel HDAC inhibitor demonstrated to enhance fear conditioning^86^, was included though it was not present in the library. All compounds are retested at 10 μM except crebinostat at 1 μm. Lithium’s effect at 1 mM was set to 100%. **e** Re-test of other novel modulators (10 μM), some of which are involved in regulation of neurotransmission. In vitro GSK3β enzymatic assay showing no direct GSK3β inhibitory activity detected for the group of compounds. Unpaired *t*-test: *0.01 ≤ *p* < 0.05, **0.001 ≤ *p* < 0.01, ***0.0001 ≤ *p* < 0.001, ****p < 0.0001. **f** Chemical structures of novel p-CRMP2^T514^ modulators that are non-lysine deacetylase inhibitors. **g** Chemical–protein interaction network presented by STITCH reveals connectivity of novel p-CRMP2^T514^ modulators to lysine deacetylation and neurotransmission.

After establishing the high-content assay with hiPSC-derived neurons and validating it with known GSK3 inhibitors, we conducted a chemogenomic screen to identify novel inhibitors of CRMP2 phosphorylation. Specifically, we tested a collection of 240 known bioactive compounds and FDA-approved drugs that were curated and generously provided by the International Rett Syndrome Foundation (Selected Molecular Agents for Rett Therapy library [SMART library]). For this screen, all compounds were initially tested at 10 μM with a 24-h treatment duration, with CHIR-99021 (10 μM) as a positive control. As shown in Fig. 4a, p-CRMP2^T514^ intensity was reduced to 23.2% by 10 μM CHIR-99021 and the calculated Z′ factor for the high-content assay reached 0.6. Since our high-content image analysis pipeline included a count of the number of MAP2-positive neurons this allowed us to assess levels of toxicity of compounds as a counter screen (Supplemental Fig. 9).

As anticipated, among the primary screening hits that reduced p-CRMP2^T514^ level to <90% of the averaged signal from all wells were a diverse set of GSK3 inhibitors (Fig. 4b). In addition, we identified novel modulators, as well as neurotoxic compounds (Fig. 4b). In this dataset, all GSK3 inhibitors were identified with activity of >20% reduction of p-CRMP2^T514^ (Fig. 4c). Supplemental Table 2 summarizes the known GSK3 inhibitors found active in the p-CRMP2^T514^ assay that have been shown previously to have activity in animal behavioral models designed to probe aspect of mood neurocircuitry.

In addition to the known GSK3 inhibitors, an additional group of compounds showed a reduction to 75–90% of the DMSO level that were not known to have direct GSK3 kinase inhibitory activity that were of interest. First, we confirmed the ability of this group of compounds to reduce p-CRMP2^T514^ levels by comparing the magnitude of their effect relatively to a therapeutically relevant dose of 1 mM lithium. As expected, these tests confirmed the results from the primary screen by significantly lowering p-CRMP2^T514^ levels (Fig. 4d, e). To investigate whether the confirmed compounds whose diverse structures are shown in Fig. 4f that were not known lysine deacetylase (KDAC) inhibitors elicited their regulatory effect on p-CRMP2^T514^ levels through direct inhibition of GSK3, we tested them in a GSK3β enzymatic assay (Supplemental Fig. 10). These results showed no detectable GSK3 inhibitory activity from any of the selected compounds, suggesting therefore that they decreased p-CRMP2^T514^ without directly inhibiting GSK3β. Finally, to further investigate the relationship between GSK3β/CRMP2 and the potential targets of the compounds that reduced p-CRMP2^T514^ level to <90% of the baseline level, we used Search Tool for Interactions of Chemicals (STITCH, http://stitch.embl.de/) to create a graphical visualization of the underlying protein-chemical interaction network. The resulting interaction network connected the p-CRMP2^T514^ modulators identified in our screen within three main submodules: GSK3 signaling, lysine deacetylation, and neurotransmission (Fig. 4g). This computational network analysis using STITCH revealed mechanistic relationships between compounds that were able to reduce p-CRMP2^T514^ levels in human neurons and helped select ones for further investigation for their ability to mimic or enhance the effect of lithium in vivo in behavioral assays.

### Attenuation of hyperlocomotion in a lithium-responsive behavior test by novel p-CRMP2^T514^ suppressors

Several behavioral paradigms have been used as clinically-predictive of treatment response even though they cannot truly replicate a complex, polygenic human psychiatric condition. The amphetamine-induced hyperlocomotion (AIH) assay has historically been used to assess the anti-manic-like activity of pharmacological agents, including the series of compounds represented by JB1121(*ent-2*)/BRD3937 shown to reduced p-CRMP2^T514^ levels in our human neuronal assays^6,66,70–72^. In the AIH assay, *d*-amphetamine inhibits dopamine and other monoamine transporters, resulting in increased extracellular striatal dopamine and behavioral hyperactivity in the animals^7^. Methamphetamine may also modulate GSK3β signaling in the nucleus accumbens^73^, and our recent evidence suggests that methamphetamine increases CRMP2 phosphorylation in both rodent and human neurons^15^. To select p-CRMP2 inhibitory compounds for testing in the AIH assay, we focused on compounds without measurable direct GSK3β inhibitor activity or reported KDAC (HDAC) inhibitor activity since, as shown in our previous studies^15^, we have already demonstrated that selective Class I HDAC inhibition is capable of attenuating locomotion in the AIH test and to have antidepressant-like activity in both the forced swim test and social defeat paradigm^74,75^. On the basis of these criteria and compound availability we selected ropinirole, oxiracetam, huperzine A, sulpiride, NNC-711, forskolin, ganaxolone for in vivo behavioral testing after acute, systemic dosing either alone or in combination with lithium.

**Fig. 5:**
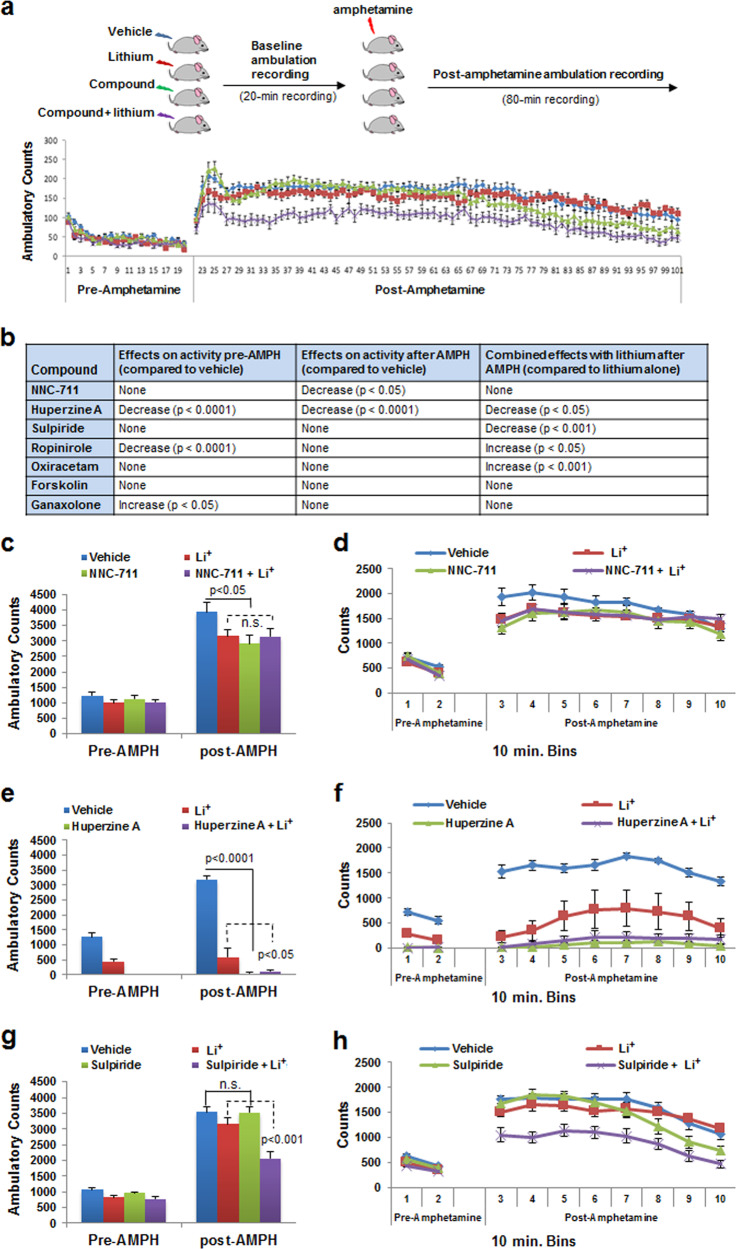
Attenuated mood-related behaviors by novel p-CRMP2^T514^ suppressors. **a** Paradigm of amphetamine-induced hyperlocomotion (AIH) mouse model. One representative recording is shown. Mouse ambulation pre-amphetamine as well as post-amphetamine exposure was recorded as a control. Effects of treatment with vehicle (blue), Li^+^ (red), compound (green), or compound plus Li^+^ (purple) on mouse ambulation pre-methamphetamine and post-methamphetamine exposure are shown as average (bar graphs, **c**, **e**, **g**) and time course showing 10-min intervals (line graphs, **d**, **f**, **h**). Two-way ANOVA’s were used to analyze the data from 32 C57BL/6J mice presented in bar graphs with the between-subjects factors Li^+^ (vehicle or Li^+^) and compound (vehicle or compound). **b** Summary of test results for the selected compounds in the AIH assay. A number of functional behavioral response patterns were observed. **c**, **d** NNC-711 decreased hyperlocomotion post-AMPH administration to the same degree as lithium and no additive or synergistic effect was detected with Li^+^, suggesting that it operated in the same pathway and/or employed a shared mechanism hence not augmented by its combination with lithium. **e**, **f** Huperzine A also decreased hyperlocomotion post-AMPH administration to the same degree as lithium, and further decrease on combination with lithium. Huperzine A was detected to decrease ambulation prior to amphetamine administration, however, its therapeutic specificity for this system is supported by observing that another compound, ropinirole, which also decreased activity pre-amphetamine, did not do so post-amphetamine. **g**, **h** Sulpiride decreased post-AMPH hyperlocomotion when combined with Li^+^ but not on its own, suggesting it may work in a parallel pathway. Hence NNC-711 and huperzine A might be viewed as a substitute for lithium while sulpiride might be thought of as an adjunct to lithium.

For these in vivo behavioral assessments, AIH activity of C57BL/6J mice pre-treated with vehicle, test compound, lithium, or lithium in combination with the test compound (Fig. 5a) was compared using a two-way ANOVA between-subjects analysis among 64 mice. Mouse ambulation pre-amphetamine was also recorded as a control for potentially confounding effects on baseline locomotion. Overall, a number of behavioral response patterns emerged as summarized in Fig. 5b. Notably, NNC-711 and huperzine A decreased hyperlocomotion post-amphetamine administration to the same degree as lithium (Fig. 5c–f). NNC-711 did not have an additive or synergistic effect with lithium (Fig. 5c, d) suggesting that it operated in the same pathway and/or employed a shared mechanism not augmented by its combination with lithium. Further decreased locomotion was observed for huperzine A combined with lithium (Fig. 5e, f). Although huperzine A decreased ambulation prior to amphetamine administration, another compound, ropinirole, which also decreased activity pre-amphetamine, did not do so post-amphetamine. In contrast, sulpiride decreased AIH when combined with lithium without affecting baseline activity or AIH levels when tested independently (Fig. 5g, h). Further testing of the effect of sulpiride in additional experiments applying between-group comparison revealed similar results (Supplemental Fig. 11). Finally, while forskolin and ganoloxone exhibited no effect on AIH, surprisingly, ropinirole and oxiracetam reversed the effect of lithium on AIH (Fig. 5b). As a final test, we wondered whether strain specificity could be contributory. To test this possibility, the effects of either huperzine A, ropinirole, or oxiracetam on AIH were further examined in DBA/2J mice. The effects of these compounds in this strain were similar in each case to the effects observed with C57BL/6J mice (Supplemental Fig. 12). Overall, our results demonstrate that a subset of compounds identified by their ability to suppress p-CRMP2^T514^ levels in human neurons recapitulated or enhanced the effects of lithium on AIH.

## Discussion

This study, in combination with our previous findings using hiPSC-derived neurons from healthy controls and BD patients^15^, demonstrates that lithium treatment at physiologically relevant levels decreases the abundance of the inactivated form of CRMP2, p-CRMP2^T514^, without decreasing total and unphosphorylated CRMP2. We previously reported that the “set-point” for the p-CRMP2^T514^:CRMP2 ratio was abnormally high in lithium-responsive BD patients (compared with non-lithium-responsive BD patients as well as patients with other psychiatric and neurological disorders), but was normalized by lithium when it decreased p-CRMP2^T514^ levels^15^. Normalization of CRMP2 activity was also accompanied by an improvement in dendritic morphometrics and neuronal activity^15^. With the identification here of multiple GSK3 inhibitors also having this effect, many of which have anti-manic-like and/or antidepressant-like activity in rodent models (Supplemental Table 2), this correlation further establishes p-CRMP2^T514^ as a robust marker of GSK3β inhibition in human neurons.

The development of GSK3 inhibitors for use in humans as therapeutics has been hampered by the lack of knowledge of relevant neural substrates involved in mediating behavior. Our findings overall support the notion that CRMP2 may be a relevant neural substrate mediating the behavioral effects of GSK3 inhibition. By identifying molecules that can increase CRMP2 function by reducing its phosphorylation by GSK3, it is possible that the compounds discovered here may have a beneficial effect in disorders where a loss of CRMP2 function has been implicated, including Alzheimer’s disease^27^, multiple sclerosis^28,29^, and Rett syndrome^30,31^. In addition, our recent studies have also found uniquely elevated levels of p-CRMP2^T514^ in BD patient-specific hiPSC-derived neurons which is reduced to normal by lithium^15^.

Lithium, while effective, is not an ideal drug: it has many undesirable adverse effects (e.g., renal and endocrine), as well as a very narrow therapeutic index. A more specific and faster-onset pharmacologic treatment for BD would be desirable. Here, we significantly extend our previous finding that lithium regulates p-CRMP2^T514^ levels in hiPSC-derived NPCs and neurons to identify functional mimetics of lithium. We performed an unbiased phenotypic screen of a chemogenomic library which recovered both known GSK3 inhibitors as well as novel modulators of p-CRMP2^T514^ that did not directly modulate GSK3β kinase activity as measured using recombinant human GSK3β activity in vitro. Some of these compounds are FDA-approved drugs, which means they might be readily tested in clinical trials and re-purposed for use as BD therapeutics.

Analysis of the targets of the p-CRMP2^T514^ suppressing compounds using STITCH suggested a common property of modulating lysine deacetylases (KDACs) among many of the active compounds. While selective Class I KDAC inhibitors are capable of attenuating AIH and having antidepressant-like activity in the forced swim test, as well as in social defeat paradigms^74,75^, the relevant neural substrates mediating these behavioral effects have not been elucidated. While the acetylation of GSK3β protein (Lys205)^76^ and three acetylation sites on CRMP2 (Lys472, Lys520, Lys525, www.phosphosite.org) have been reported, other factors could also mediate the effect of these KDAC inhibitors on p-CRMP2^T514^. Since it is known that protein phosphatases can form complexes with HDACs^77,78^, we speculate that PP2A phosphatase is actively counteracting CRMP2 phosphorylation by GSK3β and this regulation is enhanced upon KDAC inhibition. Consequently, it would be interesting in the future to test whether KDAC inhibitors augment lithium’s effect in vivo in animal behavioral tests in a manner dependent upon PP2A phosphatase activity towards GSK3β and its related interactor AKT that governs the Ser9 inhibitory phosphorylation^6,8,56^.

A major consideration in the utilization of high-content screening using stem cell derivatives for drug discovery in human disease is developing a biologically-relevant secondary assay toward validating the relevance of candidate compounds. Toward this end, we subjected candidate compounds identified in our ex vivo cellular assays to in vivo behavioral testing. Overall, the in vivo test results did reveal potential candidates with relevant functional outcomes in terms of attenuating AIH, a behavioral test that has been widely used as a surrogate system to probe the pharmacology and neurobiology of dopamine-mediated behavior and lithium-responsiveness. However, the behavioral effects were not completely predictable based solely on the biochemical activity toward CRMP2 in hiPSC-derived neurons (Fig. 5b). Multiple potential reasons for this discrepancy exist, including suboptimal in vivo pharmacological properties at the dose and time chosen for analysis, the potential contribution of other cell types affected in vivo not present in our hiPSC-derived neuronal cultures, and variation in selectivity between competing targets due to differences of the doses used in cells versus in vivo.

Of note, different degrees of effects of lithium on ambulation prior to amphetamine administration were observed in separate experiments even within the same inbred mouse strain background. The observation seems most likely due to variation in penetrance of the lithium response between individual mice despite otherwise genetic homogeneity. For each experiment, the pre-amphetamine baseline recordings served internal controls for post-amphetamine ambulation for testing effects of lithium and other compounds. Therefore, our conclusions are independent to the variance of lithium effects observed for separate experiments.

Of the p-CRMP2^T514^ suppressors identified that were profiled for in vivo activity, NNC-711, a reported blocker of GABA uptake via inhibition of SLC6A1 (solute carrier family 6 member 1; (GAT1))^79^, attenuated AIH when tested independently, but showed no additive effect when tested in combination with lithium (Fig. 5c, d). Such a profile suggests a redundant or noncomplementary mechanism to that of lithium, which would require therapeutic development as an alternative to lithium. Alternatively, if the mechanism of CRMP2 phospho-regulation via the compound was distinct from that of lithium, there would be the possibility of an additive or synergistic effect, suggesting promise as a clinical augmentation strategy. This was suggested by the effects of sulpiride on behavior when combined with lithium (Fig. 5g, h). Indeed, in humans, utilization of the lowest effective dosage of lithium is beneficial toward reducing long-term side effects and prolonging the benefit of the therapy. If compounds can be identified and substantiated to improve the benefit of a lower dosage of lithium, this would might not only make the drug safer but could also broaden the clinical population who can be more responsive to lithium treatment—an achievement that would be particularly significant given that use of lithium is seen as an effective way to reduce suicidal tendencies in BD patients.

One interesting, non-direct GSK3 p-CRMP2^T514^ suppressor identified by our screen was the natural product huperzine A. This sesquiterpene alkaloid has been under investigation as a disease-modifying drug for Alzheimer’s disease and other forms of dementia due to its ability to enhance cognition and its neuroprotective activities^80^. At the molecular level, huperzine A has been shown to have acetylcholinesterase inhibitory activity and to be an antagonist of N-methyl-*D*-aspartate (NMDA) receptors^81^. In addition, consistent with our findings of decreased p-CRMP2^T514^, huperzine A has been shown to increase levels of inhibitory GSK3^α^^S21/βS9^ phosphorylation and cause accumulation of β-catenin in brains of APPswe/PS1dE9 double transgenic mice and in cultured human neuroblastoma cells^82^. Correlated with these changes, huperzine A treatment increased APP processing leading to decreased levels of pathological forms of Aβ^82^. Similar results in terms of increased GSK3β^S9^ phosphorylation have also been reported with in vivo administration of huperzine A and analogs to 3xTg-AD mice containing transgenes expressing three mutations associated with familial Alzheimer’s disease (*APP* Swedish, *MAPT* P301L, and *PSEN1* M146V)^83^. In the AIH test, huperzine A decreased AIH alone and showed additive or synergistic effect with lithium (Fig. 5e, f). Although, these results might be seen as being confounded by the decreased baseline activity before amphetamine injection, another p-CRMP2^T514^ suppressor, ropinirole, which also decreased activity pre-amphetamine, had no effect on locomotory activity post-amphetamine treatment. Nevertheless, further testing huperzine A at lower doses is advisable to eliminate the chance of undesirable sedation and motor effects.

While this manuscript was in preparation, replicating earlier studies from the Caron laboratory using conditional Cre-recombinase expressing mice^84^, recent studies from Kim et al. using CRISPR/Cas9-mediated genome engineering demonstrated that AIH responses were dependent on GSK3β activity specifically in dopamine D2 receptor (D2R)-expressing medium spiny neurons (MSNs) that play a critical role in mediating neurotransmission within the indirect pathway of the basal ganglia circuit, but not dopamine D1 receptor (D1R)-expressing MSNs within the direct pathways^85^. In these same studies, a double knockout of GSK3β and CRMP2 in D2R-expressing MSNs was shown to prevent the knockout of GSK3β from attenuating AIH. Correlated with these behavioral changes in the CRMP2 knockout was an observed decrease in dendritic complexity and spine density of MSNs in the striatum, similar to our previous report^15^. Taken together, these observations further support the conclusion that CRMP2 plays a critical role in regulating the neurocircuitry that may be involved in affective behavioral control.

In summary, the global phosphoproteomic data and novel methodology implemented in our chemogenomic screen along with the compounds discovered here as functional mimetics of lithium—reducing p-CRMP2^T514^ levels and demonstrating efficacy in a mouse behavioral test of dopaminergic signaling—provide new cellular and molecular tools to dissect the role of neuronal GSK3 and CRMP2 signaling and investigate potentially therapeutically relevant mechanisms of lithium action. The extension of these assays to include NPCs and neurons from BD patient hiPSCs will enable ex vivo studies of lithium-sensitive signaling pathways that may help identify novel targets and leads for the development of more specific and safer novel therapeutics for BD and other neuropsychiatric disorders.

## Supplementary information

Zhao_CRMP2 manuscript_Supplemental Material

Zhao_CRMP2 manuscript_Supplemental Table
