# Supplementary material for: Discovery of suppressors of CRMP2 phosphorylation reveals compounds that mimic the behavioral effects of lithium on amphetamine-induced hyperlocomotion
Source: Transl Psychiatry. 2020 Feb 24;10:76. doi: 10.1038/s41398-020-0753-6 (PMC7039883; doi:10.1038/s41398-020-0753-6)
Supplement: Supplementary file 1 — Zhao_CRMP2 manuscript_Supplemental Material [file 41398_2020_753_MOESM1_ESM.docx]

**Supplemental Information**

**
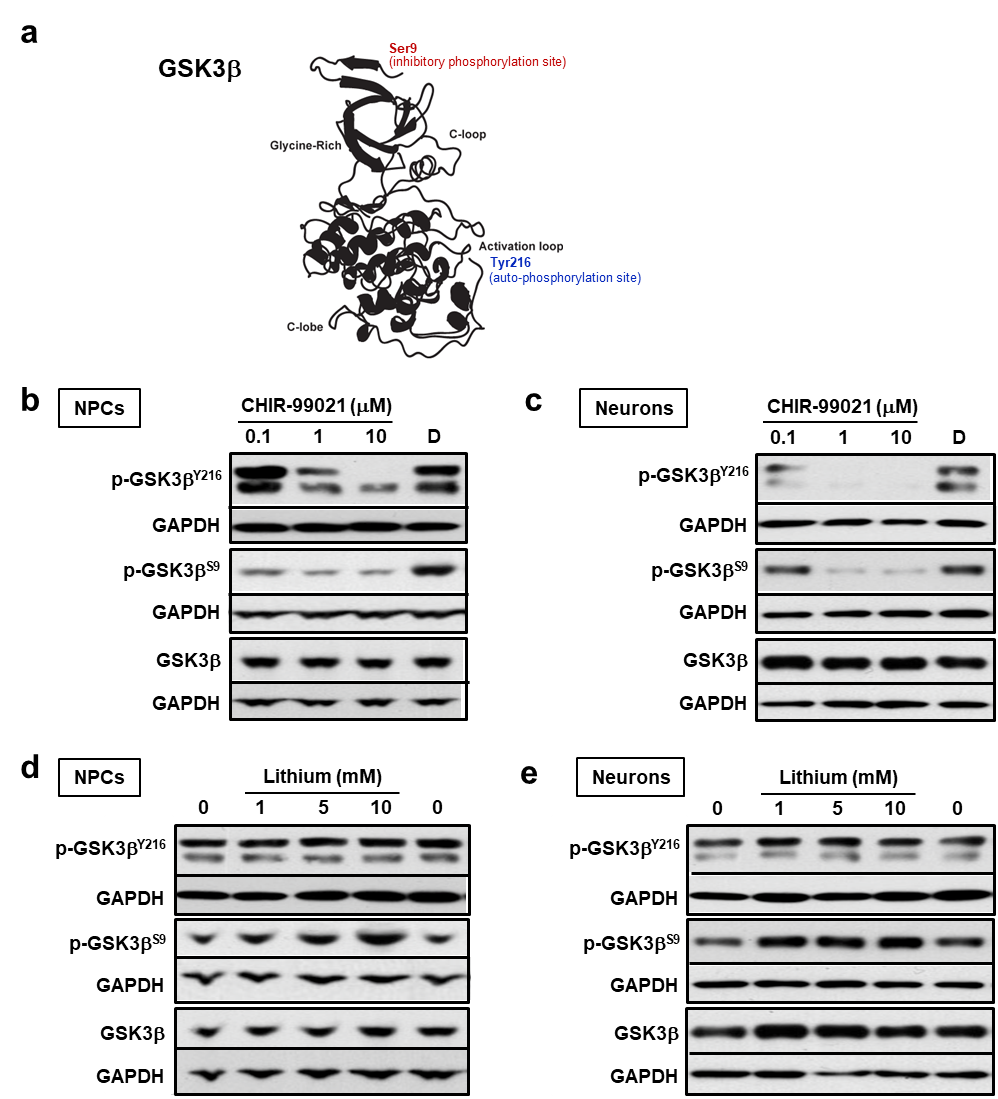
**

**Supplemental Figure 1. Western blot analysis of the effect of CHIR-99021 and lithium on p-GSK3β^Y216^ and p-GSK3β^S9^. (a)** Schematic illustration of GSK3β structure indicating the approximate locations of two important phosphorylation sites of the enzyme. GSK3β is constitutively activated by autophosphorylation at Tyr216 and inactivated by phosphorylation at Ser9**. (b)** Dose-dependent decrease of p-GSK3β^Y216^ and p-GSK3β^S9^ upon the treatment of CHIR-99021 in NPCs. Total GSK3β is not changed. **(c)** Dose-dependent decrease of p-GSK3β^Y216^ and p-GSK3β^S9^ also detected in post-mitotic neurons. **(d)** Western blots showing dose-dependent increase of p-GSK3β^S9^ for lithium treatments at 1, 5, and 10 mM whereas no change was detected for p-GSK3β^Y216^. **(e)** Lithium treatment of neuronal cultures at three concentration (1, 5, and 10 mM) showed an almost saturating effect with 1 mM lithium of increased p-GSK3β^S9^. No change was detected for p-GSK3β^Y216^.

**
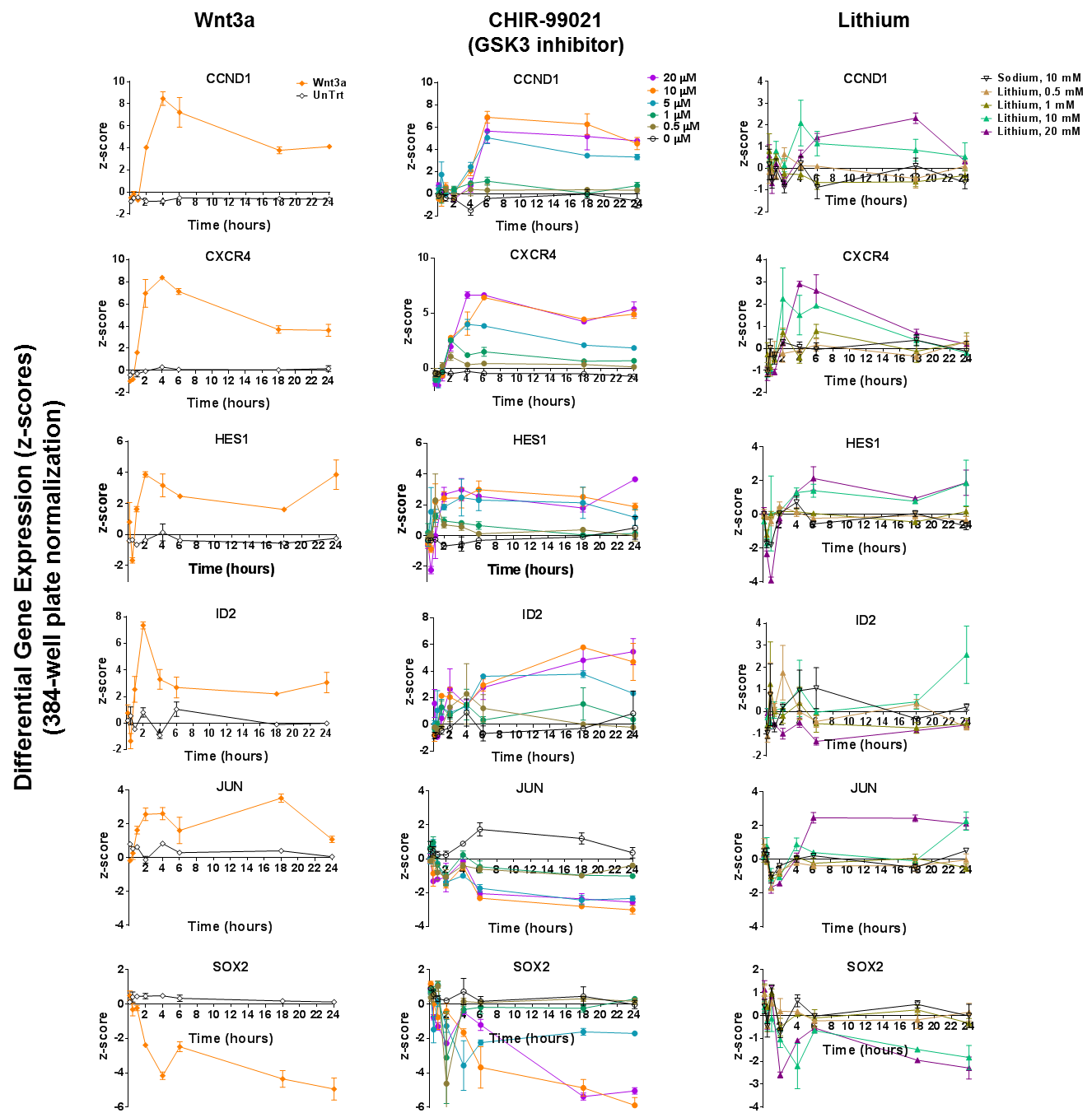
**

**Supplemental Figure 2.** **Expression profiles of WNT signaling-regulated genes by Wnt3a, CHIR-99021 and lithium treatments.** mRNA expression profiles of six WNT signaling-regulated genes were generated by L1000 gene expression profiling assay. mRNA expression levels are expressed as z-scores which were calculated by normalizing across the 384-well microplate. Error bars display standard error of the mean (SEM) of 3 biological replicates. Wnt3a treatment was carried out in the form of EC_25_ Wnt3a-conditioned media [1] with untreated (UnTrt) as control. Five doses of CHIR-99021 (0.5, 1, 5, 10, 20 μM) with DMSO (0 μM) as control and four doses of lithium (0.5, 1, 10, 20 mM) with 10 mM sodium as control were used for treatments. The six WNT genes (*CCND1, CXCR4, HES1, ID2, JUN, and SOX2*) are among 978 landmark genes directly measured by L1000 assay. Dose-dependent effects were detected for CHIR-99021 and lithium. The time-course measurements showed the expression of these WNT genes peaking at different time points, suggesting the dynamic expression of different genes in human NPCs in response to WNT pathway activation. The profiles show that CHIR-99021 and lithium regulate the expression of these genes in a similar fashion to Wnt3a with the exceptions that CHIR-99021 down-regulated *JUN*, which was up-regulated by Wnt3a, and lithium did not show strong up-regulation of *ID2*.

**
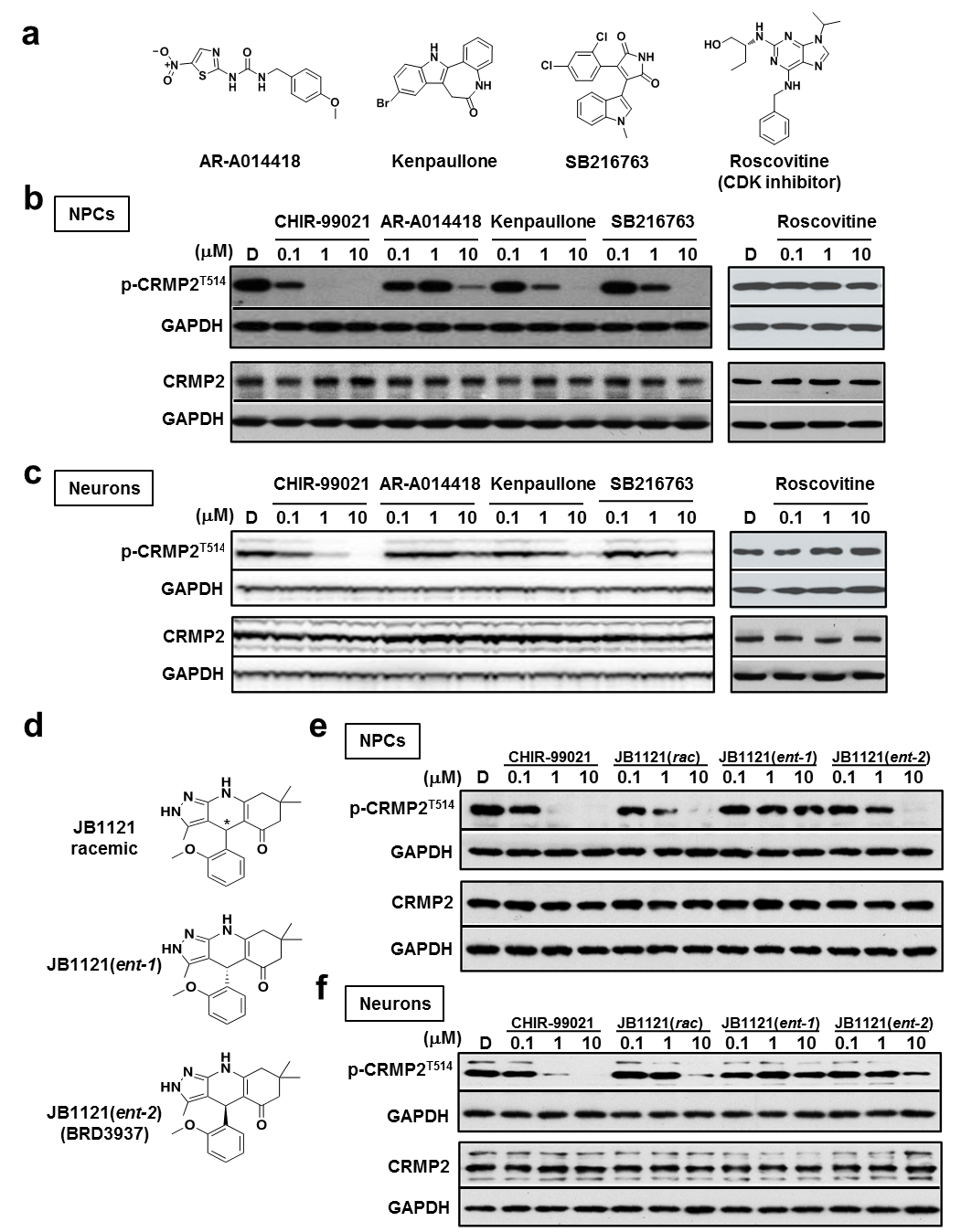
**

**Supplemental Figure 3. Prototypical and novel GSK3 inhibitors effectively reduce phosphorylation of CRMP2 at Thr514 by Western blot analysis.** **(a)** Chemical structures of diverse GSK3 and CDK inhibitors used to probe CRMP2 phosphorylation. GSK3 inhibitors with a range of inhibitory activities on GSK3 were tested for their ability to reduce p-CRMP2^T514^ levels in both NPCs **(b)** and neurons **(c)**. Dose-dependent reduction of p-CRMP2^T514^ were observed for GSK3 inhibitors, whereas no reduction from roscovitine, an inhibitor of CDK5. **(d)** Chemical structures of a novel class of GSK3 inhibitors. In NPCs **(e)** and neurons **(f)** JB1121(*ent-2*)/BRD3937 but not JB1121(*ent-1*) reduced p-CRMP2^T514^.

**
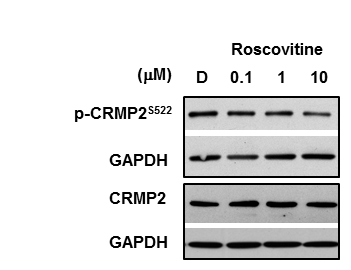
**

**Supplemental Figure 4.** Treatment of roscovitine, a CDK5 inhibitor, has no significant effect on p-CRMP2^S522^ level. CDK5 is known to phosphorylate CRMP2 at Ser522, priming it for subsequent phosphorylation by GSK3β at sites including Thr514.

**
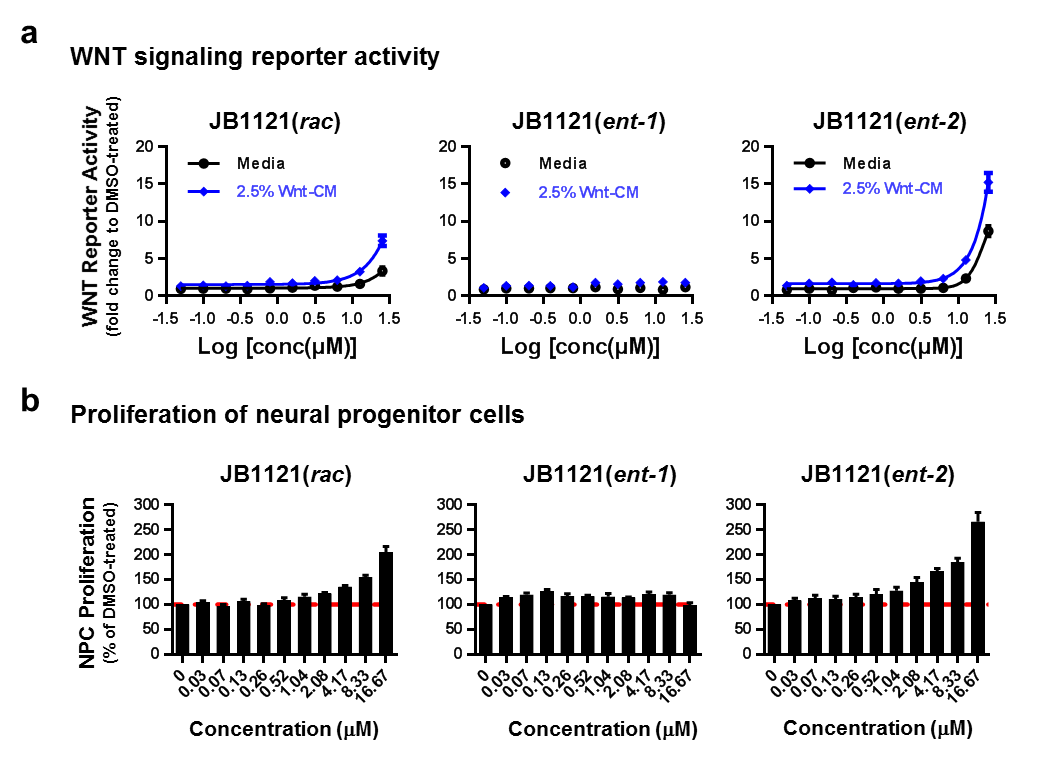
**

**Supplemental Figure 5.** (**a**) The JB1121 series of GSK3 inhibitors were tested for activation of canonical WNT signaling in NPCs measured by TCF/LEF-mediated transcription. A stable NPC line expressing TCF/LEF-luciferase gene was used. Dose response tests (10-points) were conducted in the absence or presence of 2.5% Wnt3a-condition media (Wnt-CM). Three biological replicate experiments were performed. Each data point represents the mean of quadruplicate measurements in one biological replicate. Error bars display standard error of the mean (SEM). (**b**) The JB1121 series were tested for enhancing neurogenesis with NPC proliferation detected using the CellTiter-Glo assay to measure the ATP levels from live cells. Three biological replicate experiments were performed. Each data point represents the mean of quadruplicate measurements in one biological replicate. Error bars display standard error of the mean (SEM).


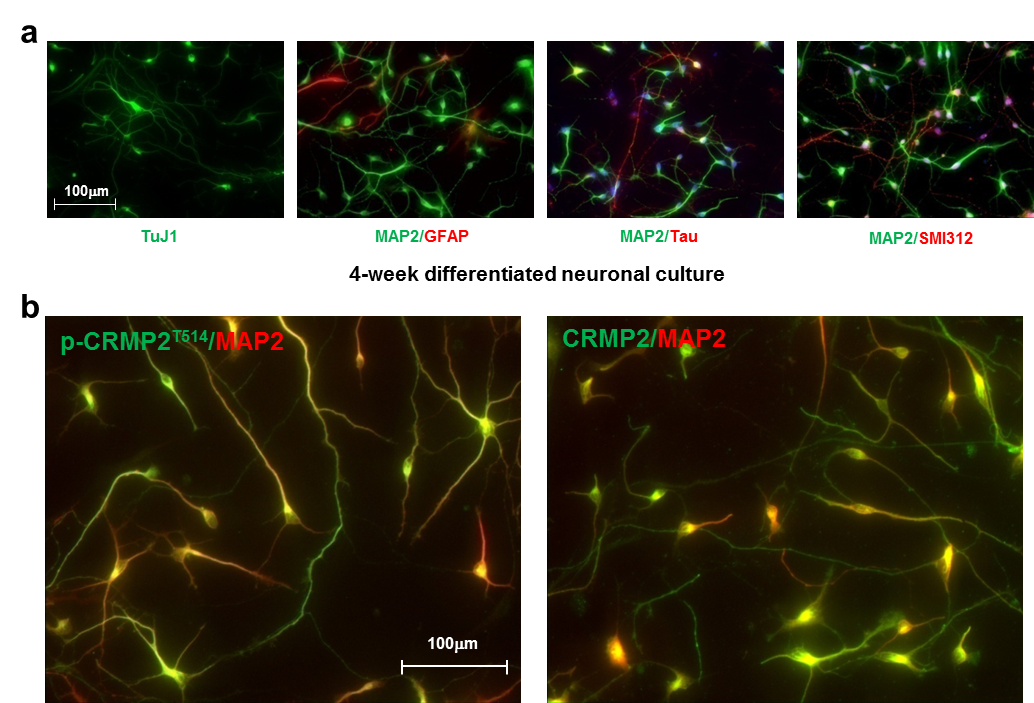


**Supplemental Figure 6.** (**a**) 4-week differentiated neuronal cultures were stained for TuJ1, MAP2 and SMI312, as well as Tau, another axonal marker. GFAP-positive astrocytes can also be found in the culture. (**b**) Immunofluorescence staining of CRMP2 or p-CRMP2^T514^ (green) co-stained with MAP2 (red). On four-week differentiated human neurons, CRMP2 and p-CRMP2^T514^ appear on all neurites and soma.


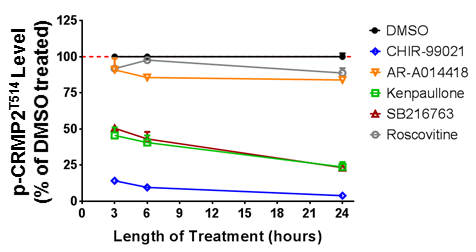


**Supplemental Figure 7. Robustness of the high-throughput phenotypic assay for modulators of p-CRMP2^T514^.** Reduced p-CRMP2^T514^ was detected at 3-hr treatment from 10 μM CHIR-99021, Kenpaullone and SB216763.

**
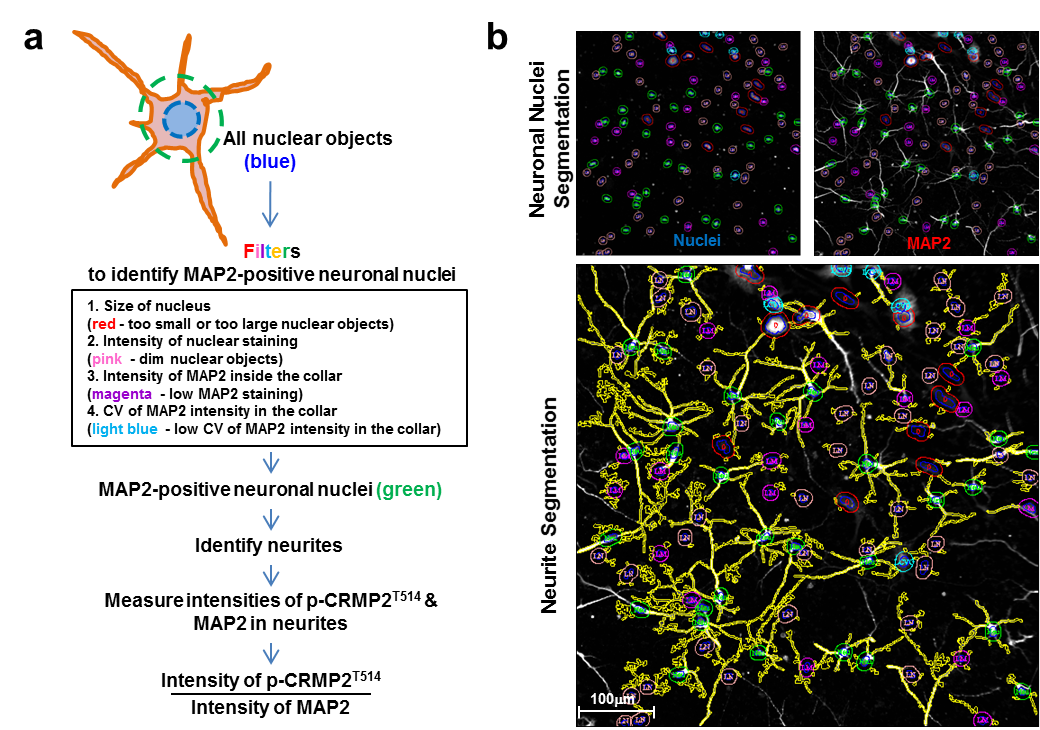
**

**Supplemental Figure 8. Custom image analysis pipeline. (a)** High-content image analysis (HCA) algorithm to measure p-CRMP2^T514^ intensity normalized to MAP2 intensity from neurites of single neuron. Filters were applied to identify MAP2-positive neurons in heterogeneous culture. Neurites were traced out from nuclear objects. Measured p-CRMP2^T514^ and MAP2 intensities in neurites of MAP2-positive neurons were used to evaluate p-CRMP2^T514^-reducing effect. (**b**) Segmentation of images illustrating the HCA algorithm in **a**. All nuclear objects are circled in blue. Red, pink, magenta and light blue circle nuclear objects excluded. Green circle with Neu denotes real MAP2-positive neurons.


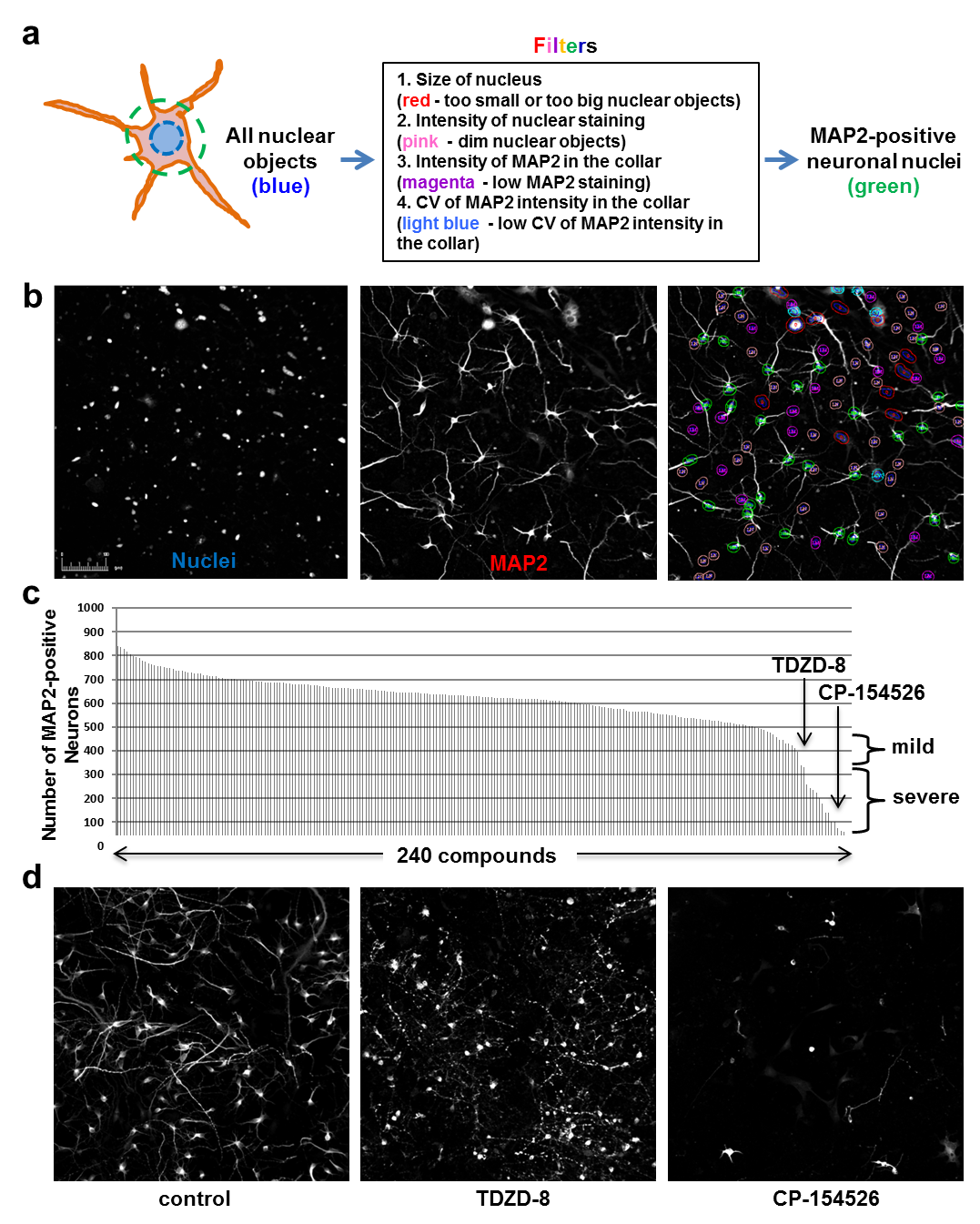


**Supplemental Figure 9. High-content analysis of MAP2 immuno-stained neuronal culture identified neurotoxic compounds present in the library.** High-throughput image acquisition was facilitated by automated confocal microscope IN Cell Analyzer 6000. 6 fields of images were taken from each well of 96-well plates with 20x objective. Image analysis was performed with Investigator software coupled with the instrument. (**a**) Image analysis pipeline for high-content analysis to identify MAP2-positive neuronal nuclei. (**b**) Segmentation of images illustrating the high-content analysis algorithm in **a**. All nuclear objects are circled in blue. Green circle with Neu denotes counted MAP2-positive neuronal nuclei. (**c**) Identification of neurotoxic compounds by high-content analysis from 240 compounds used in the screen. A group of compounds severely damaged neuronal culture health; a group showed mild effect. (**d**) Representative images showing neuronal cultures with normal, damaged, and severely damaged appearance.

**
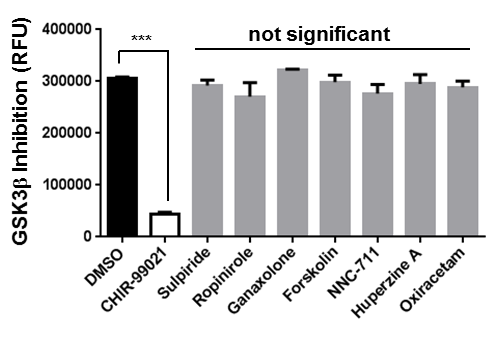
**

**Supplemental Figure 10.** *In vitro* GSK3β enzymatic assay showing no direct GSK3β inhibitory activity detected for the group of re-tested compounds.

**
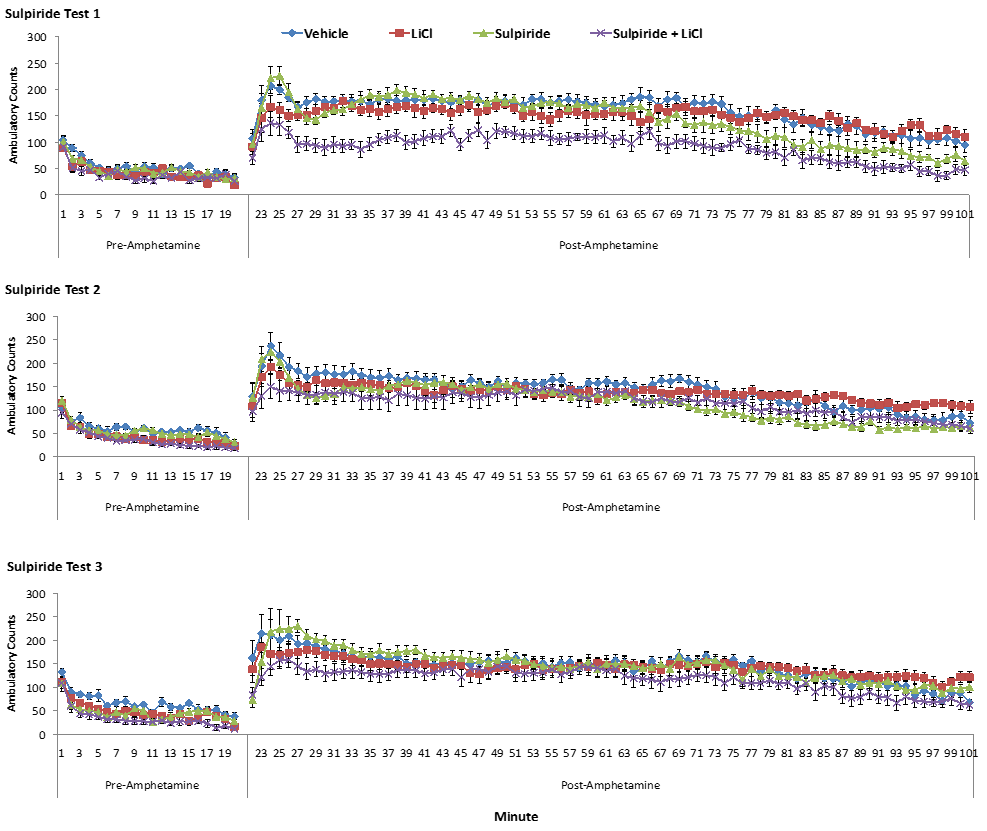
Supplementary Figure 11:**  **Between-group comparison of effects of Sulpiride.** Time course in minutes of ambulation of mice pre- and post-methamphetamine administration in response to treatment with vehicle (blue), Li^+^ (red), Sulpiride (green), or Sulpiride plus Li^+^ (purple). Three separate experiments (6 mice per each treatment group) are shown.

**
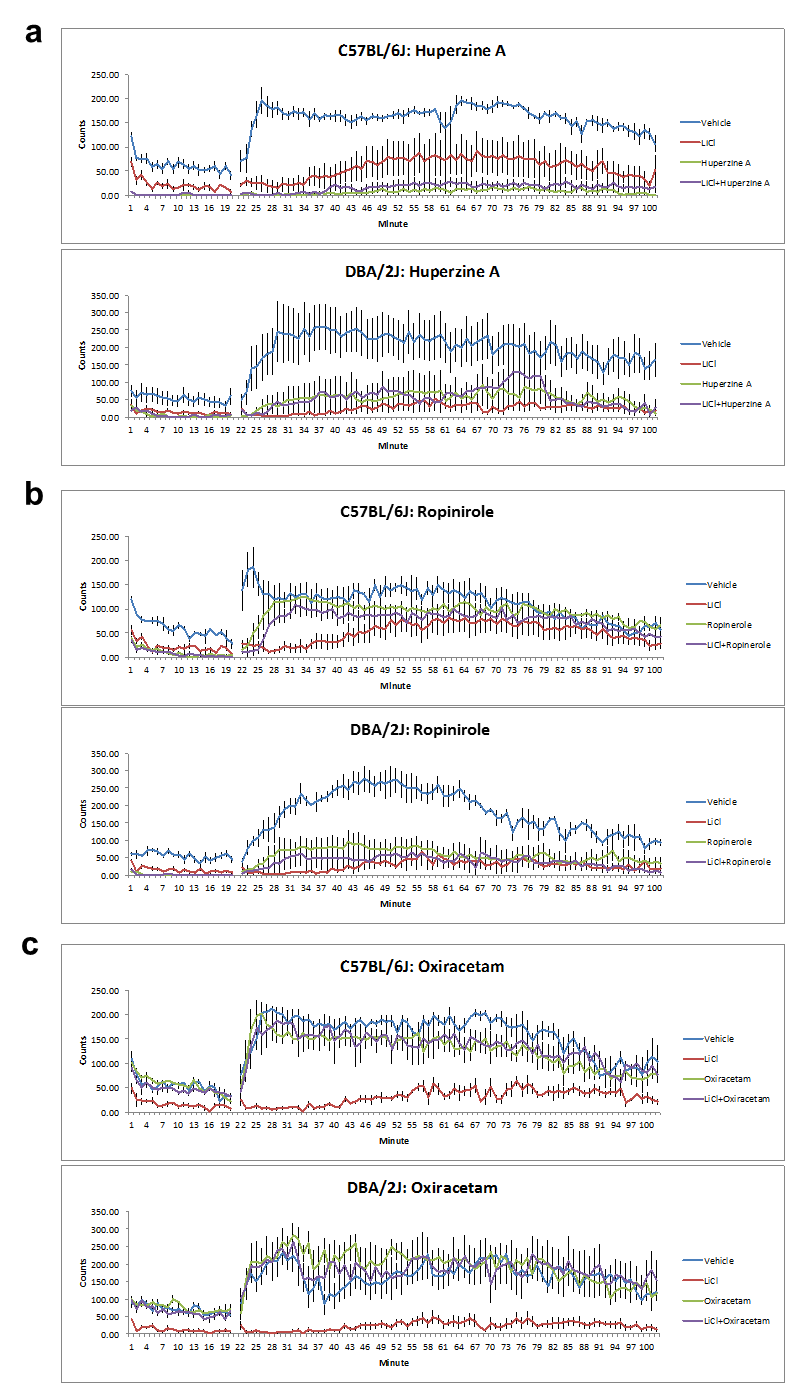
**

**Supplementary Figure 12:**  **Measurement of effects of huperzine A, ropinirole, or oxiracetam in C57BL/6J versus DBA/2J mice.** In separate experiments, 32 C57BL/6J mice and 32 DBA/2J mice were treated with vehicle (**blue**), Li^+^ (**red**), compound (**green**) or compound plus Li^+^ (**purple**), and pre-amphetamine as well as post-amphetamine ambulation was recorded.

**Supplemental Table 1. Phosphopeptides identified by SILAC phosphoproteomics upon CHIR-99021 treatment.** The complete list of high confidence, phosphopeptides identified by SILAC-MS upon treatment of hiPSC-derived NPCs with two concentrations of CHIR-99021 (1 μM or 10 μM) or vehicle control (DMSO) was generated by re-analysis of the peptide level data (massive.ucsd.edu, accession no. MSV000080975) through an updated computational pipeline and the latest version of the UniProt human proteome database, including removal of a set of 150 common laboratory contaminant proteins. In total, this new analysis identified 2,829 phosphopeptides of which 43 (in blue or brown) were significantly regulated in the CHIR-99021 (10 μM) treatment after correction for multiple hypothesis testing.

**Supplemental Table 2. GSK3 inhibitors with CRMP2^T514^ phosphorylation inhibitory activity with known behavioral activities in animal models of mood-related activity.**

| **GSK3 inhibitors** | **Reduction of amphetamine induced hyperactivity and mania** | **Anti-depressant effect (forced swim test or tail suspension test)** |
| --- | --- | --- |
| Lithium | + [2-5] | + [4-6] |
| CHIR-99021 | + [5] | + [5] |
| AR-A014418 | + [7, 8] | + [7] |
| Kenpaullone | + (alsterpaullone) [8, 9] | Not reported |
| SB216763 | + [8-10] | - [11] |
| Indirubin | + [8] | Not reported |
| Indirubin-3’-monoxime | + [9] | Not reported |
| BIP-135 | * [12] | Not reported |
| TWS-119 | Not reported | Not reported |

Note: -, negative result; * SMA (Spinal Muscular Atrophy) mouse model

**Supplemental Methods**

***L1000 Gene Expression Profiling***

NPCs in 384-well plates at 10,000 cells per well were treated with an EC_25_ level of Wnt3a-conditioned media, CHIR-99021, and lithium based upon the WNT signaling reporter gene assay. At the end of treatment, cells were lysed in lysis buffer and stored at -80°C. Plates were then processed for L1000 mRNA profiling at the Broad Institute LINCS (Library of Integrated Cellular Signatures) Center. The L1000 assay directly measures the expression of 978 landmark genes that can be extended to measure of the whole genome with a computational inference model [13]. Gene expression levels are expressed as z-scores, calculated from the entire 384-well plate. zi = (xi–median(X)) / (MAD(X)*1.4826), where X is the vector of normalized gene expression of gene x across all samples on the plate. The “median” and “MAD” represent median and median absolute deviation [MAD = median(IXi-median(X)I)], respectively.

***WNT Signaling Reporter Gene Assay***

Activities of WNT signaling stimulation were measured with WNT reporter gene stable NPC line. The assay was performed by following procedures described previously [1]. Briefly, single cell suspensions of the reporter NPCs were dispensed into pre-coated 384-well plates at 6000 cells per well in 30 μL culture media. 24 hrs later, 10 μL of media or media containing Wnt3a-conditioned media (CM) was dispensed to each well to achieve media-only or 2.5% Wnt3a-CM in final concentration, followed by compound treatment. After 24-hr treatment, cells were lysed by dispensing 15 μL of SteadyGlo reagent (Promega) and read for luminescence. Activities of WNT signaling stimulation are expressed as fold change over DMSO-treated samples.

***NPC Proliferation Assay***

NPCs from 8330-8 were expanded and seeded to pre-coated 384-well plates the same way described for WNT signaling reporter assay [1]. Cells were treated next day, and 24 hrs later, 15 μL of CellTiter-Glo reagent (Promega) was dispensed to each well. Luminescence was read on EnVision multimodule plate reader (PerkinElmer). The CellTiter-Glo assay was performed by following procedures described by the manufacturer (Promega). Proliferation of NPCs are expressed as percentage of DMSO-treated samples which are set to 100%.

***In Vitro Enzymatic Assay for GSK3β Inhibitory Activity***

Compounds were pre-incubated for 20 minutes in assay buffer (50 mM Tris, pH 7.5, 5 mM MgCl_2_, 0.01% Brij-25, 3 mM DTT) with peptide substrate (GSP-2 peptide: Tyr-Arg-Arg-Ala-Ala-Val-Pro-Pro-Ser-Pro-Ser-Leu-Ser-Arg-His-Ser-Ser-Pro-His-Gln-Ser(PO3H2)-Glu-Asp-Glu-Glu-Glu, KareBay Biochem) and enzyme GSK3β (BPS Biosciences, Human Recombinant, N-terminal GST-tag). GSK3β kinase reaction was performed with 9 μM GSP-2 peptide, 15 μM ATP, 10 nM GSK3β. Kinase activity was detected using ADP-Glo assay reagents following manufacturer’s protocol (Promega).

**Supplemental References**

1. Zhao, W.N., et al., *A high-throughput screen for Wnt/beta-catenin signaling pathway modulators in human iPSC-derived neural progenitors.* J Biomol Screen, 2012. **17**(9): p. 1252-63.

2. Cox, C., et al., *Lithium attenuates drug-induced hyperactivity in rats.* Nature, 1971. **232**(5309): p. 336-8.

3. Davies, C., et al., *Lithium and alpha-methyl-p-tyrosine prevent "manic" activity in rodents.* Psychopharmacologia, 1974. **36**(3): p. 263-74.

4. Gould, T.D., et al., *Beta-catenin overexpression in the mouse brain phenocopies lithium-sensitive behaviors.* Neuropsychopharmacology, 2007. **32**(10): p. 2173-83.

5. Pan, J.Q., et al., *AKT kinase activity is required for lithium to modulate mood-related behaviors in mice.* Neuropsychopharmacology, 2011. **36**(7): p. 1397-411.

6. O'Brien, W.T., et al., *Glycogen synthase kinase-3beta haploinsufficiency mimics the behavioral and molecular effects of lithium.* J Neurosci, 2004. **24**(30): p. 6791-8.

7. Gould, T.D., et al., *AR-A014418, a selective GSK-3 inhibitor, produces antidepressant-like effects in the forced swim test.* Int J Neuropsychopharmacol, 2004. **7**(4): p. 387-90.

8. Kalinichev, M. and L.A. Dawson, *Evidence for antimanic efficacy of glycogen synthase kinase-3 (GSK3) inhibitors in a strain-specific model of acute mania.* Int J Neuropsychopharmacol, 2011. **14**(8): p. 1051-67.

9. Beaulieu, J.M., et al., *Lithium antagonizes dopamine-dependent behaviors mediated by an AKT/glycogen synthase kinase 3 signaling cascade.* Proc Natl Acad Sci U S A, 2004. **101**(14): p. 5099-104.

10. Enman, N.M. and E.M. Unterwald, *Inhibition of GSK3 attenuates amphetamine-induced hyperactivity and sensitization in the mouse.* Behav Brain Res, 2012. **231**(1): p. 217-25.

11. Ma, X.C., et al., *Long-lasting antidepressant action of ketamine, but not glycogen synthase kinase-3 inhibitor SB216763, in the chronic mild stress model of mice.* PLoS One, 2013. **8**(2): p. e56053.

12. Chen, P.C., et al., *Identification of a Maleimide-Based Glycogen Synthase Kinase-3 (GSK-3) Inhibitor, BIP-135, that Prolongs the Median Survival Time of Delta7 SMA KO Mouse Model of Spinal Muscular Atrophy.* ACS Chem Neurosci, 2012. **3**(1): p. 5-11.

13. Subramanian, A., et al., *A Next Generation Connectivity Map: L1000 Platform and the First 1,000,000 Profiles.* Cell, 2017. **171**(6): p. 1437-1452 e17.
